# *In vivo* imaging reveals novel replication sites of a highly oncogenic avian herpesvirus in chickens

**DOI:** 10.1371/journal.ppat.1010745

**Published:** 2022-08-29

**Authors:** Isabelle Lantier, Corentin Mallet, Laurent Souci, Thibaut Larcher, Andele M. Conradie, Katia Courvoisier, Sascha Trapp, David Pasdeloup, Benedikt B. Kaufer, Caroline Denesvre

**Affiliations:** 1 INRAE, UMR1282 ISP, Centre INRAE Val de Loire, Nouzilly, France; 2 INRAE, Oniris, PAnTher, APEX, Nantes, France; 3 Institut für Virologie, Freie Universität Berlin, Berlin, Germany; 4 Veterinary Centre for Resistance Research (TZR), Freie Universität Berlin, Berlin, Germany; Oklahoma State Univeristy, UNITED STATES

## Abstract

*In vivo* bioluminescence imaging facilitates the non-invasive visualization of biological processes in living animals. This system has been used to track virus infections mostly in mice and ferrets; however, until now this approach has not been applied to pathogens in avian species. To visualize the infection of an important avian pathogen, we generated Marek’s disease virus (MDV) recombinants expressing firefly luciferase during lytic replication. Upon characterization of the recombinant viruses *in vitro*, chickens were infected and the infection visualized in live animals over the course of 14 days. The luminescence signal was consistent with the known spatiotemporal kinetics of infection and the life cycle of MDV, and correlated well with the viral load measured by qPCR. Intriguingly, this *in vivo* bioimaging approach revealed two novel sites of MDV replication, the beak and the skin of the feet covered in scales. Feet skin infection was confirmed using a complementary fluorescence bioimaging approach with MDV recombinants expressing mRFP or GFP. Infection was detected in the intermediate epidermal layers of the feet skin that was also shown to produce infectious virus, regardless of the animals’ age at and the route of infection. Taken together, this study highlights the value of *in vivo* whole body bioimaging in avian species by identifying previously overlooked sites of replication and shedding of MDV in the chicken host.

## Author summary

*In vivo* bioluminescence imaging is a powerful tool to track virus infection in the whole body of living animals. This system has been successfully used in mice, ferrets, rats and even fishes, but until now never in birds. In this study, we performed the first *in vivo* imaging assessing the spread of an important avian pathogen, the highly oncogenic Marek’s disease virus (MDV). Using a recombinant virus expressing firefly luciferase, we visualized the course of MDV infection in chicks for 14 days. The bioluminescent signal was consistent with the known kinetics and sites of dissemination of MDV, notably in feathers. With this new approach, we also discovered two novels sites of early infection and replication that may contribute to persistent virus shedding. Both novel sites represent hard skin appendages like the feathers: the beak and the skin of the feet that are covered in scales. These results were confirmed with two recombinant viruses expressing fluorescent proteins. Fifty-five years after the discovery of MDV and thanks to in vivo imaging, we provide new insights in MDV life cycle *in vivo*, highlighting the importance of bioluminescence imaging of the entire body in living animals.

## Introduction

Infectious disease research in the past relied heavily on the detection of pathogens in biopsy material or post mortem samples. However, this only allows the assessment of disease processes in small parts of the body and/or at a single time point. The discovery of luciferase enzymes was a game changer for pathogen research in infected animals. After administration of the respective luciferase substrate, luciferase enzymes emit light that can be detected using dedicated bioluminescence imaging cameras. Genetic engineering of various pathogens including viruses, bacteria and parasites facilitates the insertion of luciferase genes into their genomes. These recombinant pathogens can be subsequently used to detect the site(s) of infection in the living host in a non-invasive manner over time. This not only reveals the anatomical location of infection, but also the replication of the pathogen in specific sites and organs by measuring the intensity of the bioluminescence signal. This approach has been successfully used for a number of viruses, bacteria and parasites including herpes simplex virus 1, hepatitis B and C viruses, influenza virus and Sars-CoV-2; *Mycobacterium* spp. and *Methicillin-resistant S*. *aureus* (MRSA); and *Plasmodium berghei* (malaria), leishmania, and toxoplasma, respectively. Despite this broad body of evidence, bioluminescence imaging has mainly focused on the analysis of infections in mice, ferrets and fish [1–6], while no study to our knowledge has been reported in birds.

One important avian pathogen is Marek’s disease virus (MDV), which causes a deadly lymphoproliferative disease in chickens and causes immense economic losses in poultry industry worldwide [7, 8]. Current MDV vaccines successfully protect against clinical disease, but still permit virulent strains to infect the host, efficiently replicate and spread to the next individual. Circulation of virulent strains in vaccinated flocks poses a risk as more virulent strains can evolve as observed in the past [9]. To develop more effective vaccines, a better understanding of MDV biology and pathogenesis is needed [10]. MDV is a highly cell-associated virus, as the spread of infection within the host occurs via cell-to-cell contact. According to the current model of MDV pathogenesis [11], infection is initiated by the inhalation of infectious dust or dander from a contaminated environment [12]. In the upper respiratory tract, the infectious dust is taken up by phagocytic cells, like macrophages, dendritic cells or B cells [13], which subsequently transport the virus to lymphoid organs: the bursa of Fabricius, thymus and spleen [14]. In these organs, MDV efficiently replicates in mainly B cells and T cells until the virus establishes latency around day 10–14 post infection [11]. MDV primarily establishes latency infection in CD4+ T cells, which can be also transformed resulting in the development of deadly lymphomas [15]. Finally, lytically and/or latently infected T cells transport the virus to the feather follicle epithelium, where infectious “cell-free” virus is produced and shed into the environment starting at 2 weeks post-infection [16, 17]. To date, feather, notably the feather follicle epithelium, is the only tissue known to shed infectious virions in the environment and the unique source of MDV transmission. Lytic infection in this tissue was shown by detecting mRNA of lytic genes by reverse transcriptase quantitative polymerase chain reaction (RT-qPCR), lytic viral antigens by fluorescence microcopy and western blotting, and complete virions by electron microscopy [17–19].

In this first *in vivo* bioimaging study using experimentally infected chickens, we established the basic parameters for the imaging protocol in birds and explored the MDV infection dynamics in a spatiotemporal manner. Using a recombinant virus that encodes firefly luciferase (fLuc), we could demonstrate for the first time that MDV is not only transported to the feather follicles, but also to the beak and the skin of the feet covered with scales. This unexpected observation was confirmed using two fluorescently labeled reporter viruses by bioimaging, qPCR, infectivity assay and histology. Taken together, our study revealed a rapid spread of MDV to unexpected anatomic sites which were missed by standard sampling for more than fifty years, providing important insights into this deadly disease in chickens.

## Results

### Generation of a fLuc reporter MDV

**Fig 1:**
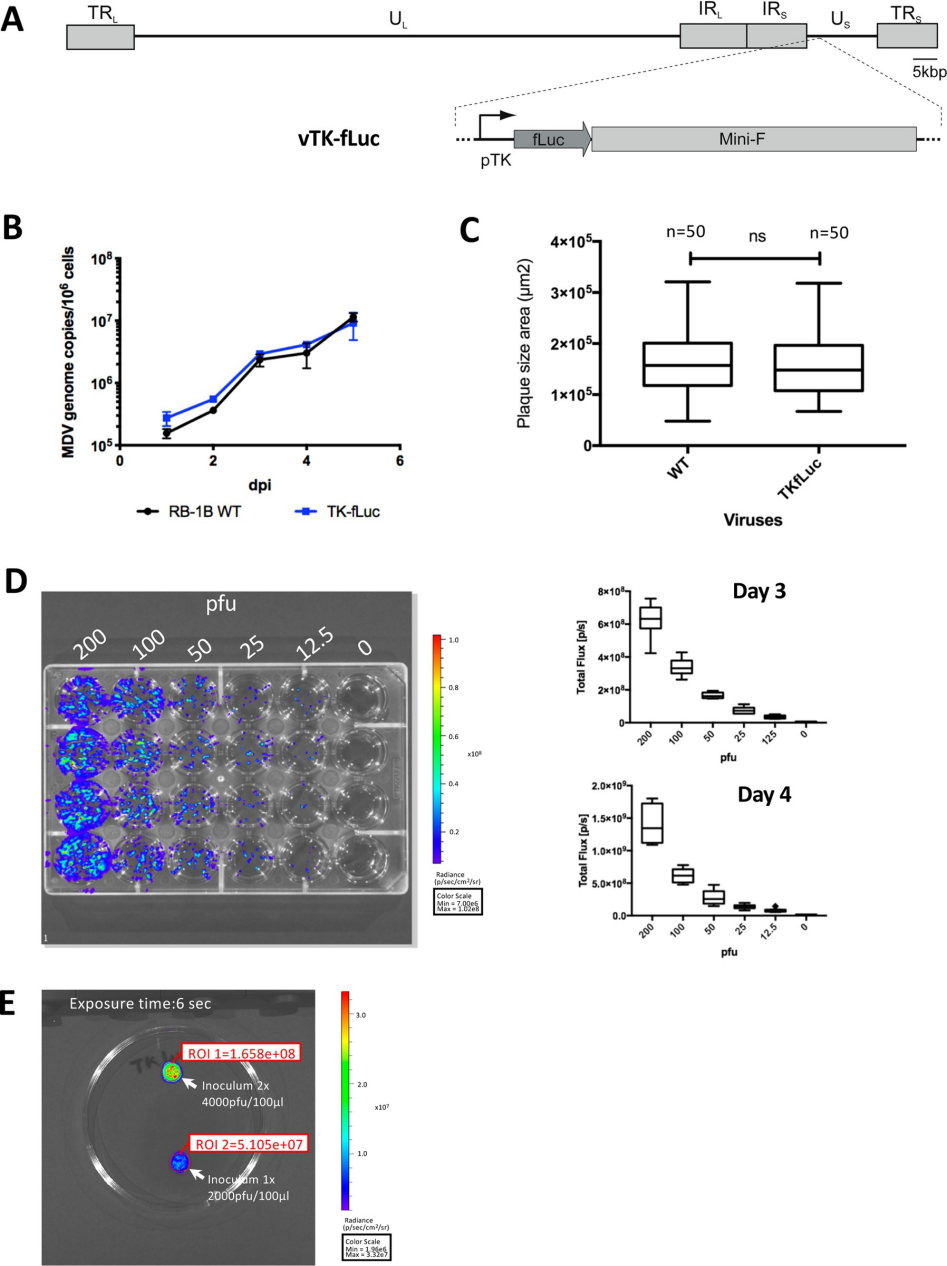
The vTK-fLuc generates a bright bioluminescence signal in cell culture and spreads as the parental virus (WT). (A) Overview of the MDV genome containing the vTK-fLuc cassette. (B) Replication was assessed by multi-step growth kinetics 1 to 5 dpi. (C) Plaque size assay. The size of fifty plaques in CESCs was measured at 4 dpi. The difference in the plaques size between the vTK-fLuc and the WT was not significant (ns; Mann-Whitney test; p-value = 0.6187) indicating that the vTK-fLuc spreads comparable to WT. (D) Assessment of dose dependence. Serial dilutions of infected cells from 12.5 pfu to 200 pfu were used to infect CESCs. The plates were imaged with the IVIS Spectrum for bioluminescence at 3 and 4 dpi. A representative image is provided (3 dpi). In each well the photon/s was quantified and plotted (left panel). Data are shown in tukey boxes (n = 8 for each infectious dose at each time point). (E) Bioluminescence of an infectious cell suspension prepared as an inoculum with 2000 and 4000 pfu per drop, mixed with D-luciferin. The results per region of interest are shown as total flux (photons/s).

To visualize virus infection *in vivo*, we generated a recombinant MDV expressing fLuc driven by the early HSV-1-TK promoter (vTK-fLuc, Fig 1A), using the bacterial artificial chromosome (BAC) system of the very virulent RB-1B strain. The bioluminescence signal of vTK-fLuc plaques was analyzed using an IVIS Spectrum imager. The signal in total flux was very intense with 1.489x10^9^ photons/second (p/s) per 100 plaques. Replication kinetics and plaque size assay revealed that the replication properties of vTK-fLuc were comparable to the parental BAC-derived rRB-1B virus (Fig 1B and 1C). To assess the dose dependency of the luciferase signal, chicken embryo skin cells (CESCs) were infected with 12.5, 25, 50, 100 and 200 plaque forming units (pfu) per well of vTK-fLuc and the luciferase signal was measured at 3 and 4 days post-infection (dpi) using the IVIS Spectrum. A dose-dependent luciferase activity was observed at both time points indicating that the luciferase signal correlates well with virus replication (Fig 1D). This prompted us to use this recombinant virus in the subsequent *in vivo* imaging approach. A strong luciferase signal of 5.1x10^7^ and 1.66 x10^8^ p/s was detected for 2000 and 4000 pfu of the vTK-fLuc inoculum used for the *in vivo* studies (Fig 1E).

### Establishment of the parameters for bioluminescence *in vivo* imaging in chickens

As there is only one report on bioluminescence imaging of uninfected chickens in the literature [20], we first determined key imaging parameters such as the auto-luminescence of feed and the feathered-body, and then validated the route and dose of D-luciferin (D-Luc) injection for fLuc detection. In all bioluminescence imaging experiments, the luminescence signal was assessed as total flux (p/s) and/or as average radiance (photons/sec/cm^2^/steradian; p/s/cm^2^/sr) for the selected regions of interest (ROI). The chicken feed exhibited a signal of 3.69x10^5^ p/s for 2g and of 2.21 x10^3^ p/s/cm^2^/sr (S1A Fig). The average auto-luminescence of White Leghorn (WL) B13 SPF chickens (5 to 8 days post-hatch) was below 3x10^3^ p/s/cm^2^/sr, with or without D-Luc injection (S1B Fig). Next, we assessed whether a subcutaneous dose of 0.150 mg D-Luc per g of body weight as recommended by the manufacturer (PerkinElmer) for bioluminescence imaging in mice was sufficient for the fLuc detection in chickens and as previously reported in newly hatched chicks [20]. For this, an anesthetized 10-day old chicken was inoculated with vTK-fLuc-infected CESCs (about 10^4^ pfu) in two different locations: intramuscularly into the breast muscle and subcutaneously in the abdomen above the cloaca. The D-Luc solution was administrated subcutaneously in the back. A bioluminescence signal was readily detectable at the two sites of MDV injection, 7 min after D-Luc injection (S1C Fig) indicating a rapid bio-availability of D-Luc. No signal was visible in other parts of the body. The signal was higher 10 min after D-Luc injection and remained stable at 15 min (S1C Fig). We thus confirmed the dose and route of D-Luc for chickens and determined 10 min after D-Luc subcutaneous injection as an optimal timepoint for imaging.

### Spatiotemporal monitoring of bioluminescence in live chickens infected with vTK-fLuc

**Fig 2:**
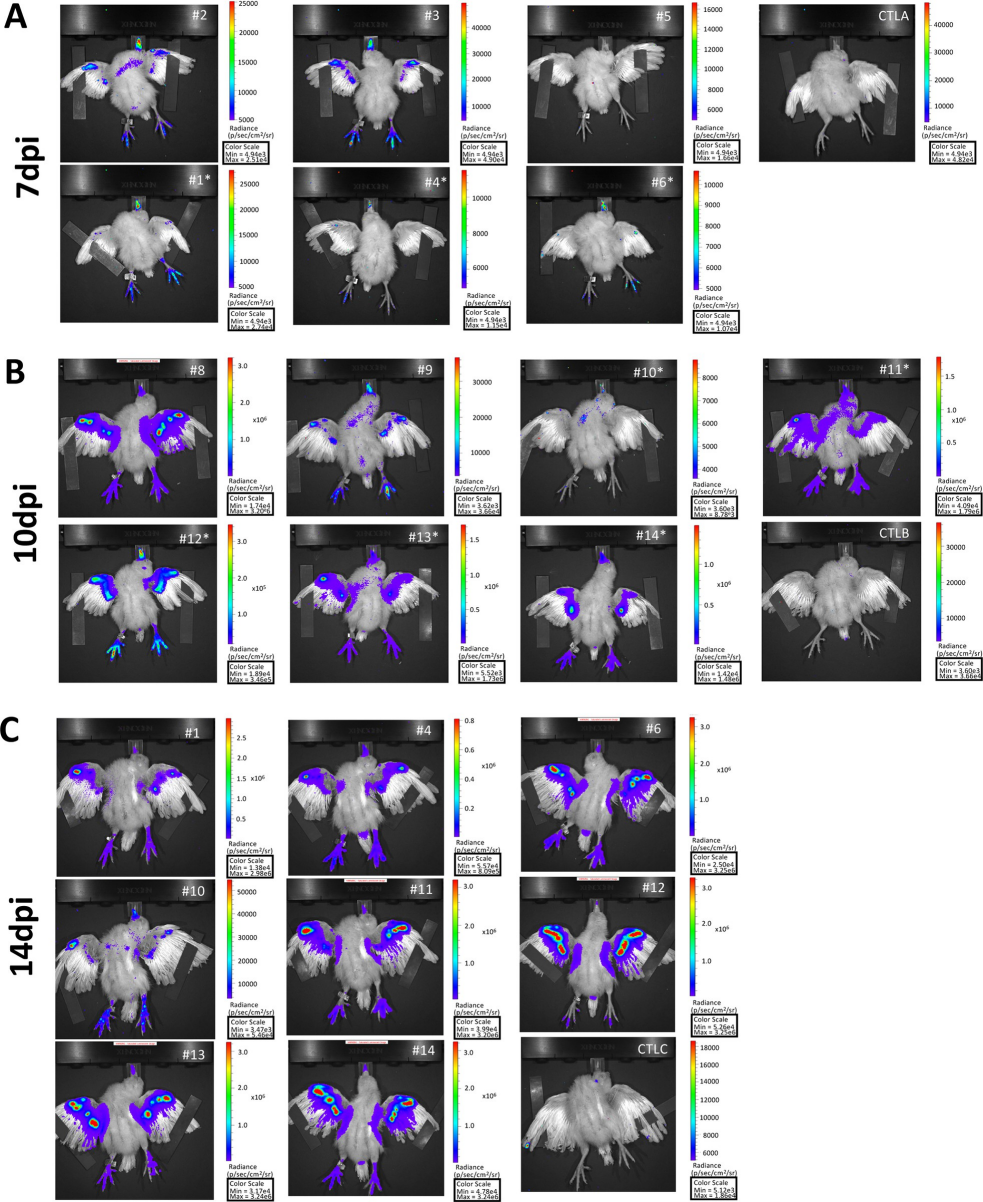
Bioluminescence *in vivo* imaging of vTK-fLuc-infected chickens. One day old-chickens were inoculated intramuscularly with 2000 pfu of vTK-fLuc. Chickens were imaged *in vivo* with an IVIS spectrum: (A) 6 chickens at 7 dpi, (B) 7 chickens at 10 dpi and 8 chickens at 14 dpi. Chickens indicated by an asterisk were kept alive after *in vivo* imaging. An image of each chicken imaged is shown. At each time point, a naive control bird of matched age (Control, CTL) was imaged for comparison (CTL A, B and C). Note that all chickens imaged at 14 dpi were imaged earlier, either at 7 or 10 dpi. Each chicken image is shown with its own radiance scale (p/s/cm2/sr) due to variations between individuals.

To visualize early MDV infection *in vivo*, we infected 13 one-day-old WL B13 SPF chickens intramuscularly with 2000 pfu of vTK-fLuc. Groups of chickens were imaged at 7, 10 and 14 dpi, 10 min after subcutaneous injection of the D-Luc substrate. At day 7, two animals (#2,3) on six that were imaged, exhibited clear signals at the base of the wing flight feathers (remiges; Fig 2A). Bioluminescent signal was detected in the upper chest, the anatomical region of the thymus, of bird#2. Surprisingly, almost all animals (except #5) exhibited a strong bioluminescence signal at the beak and feet (Fig 2A). This was a striking observation as viral tropism for these sites has never been described before. No signal was detectable in the control chickens that were imaged under the same conditions.

At 10 dpi, seven other infected chickens were imaged (#8,9,10,11,12,13,14) (Fig 2B). All chickens except one (#10) exhibited a clear luminescence signal at the base of the wing feathers as well as at the beak and the feet. Chicken #10 showed only a weak signal in the beak and on the left side of the chest. Chicken #9 had also a faint signal in the upper chest and lower abdomen, the anatomical region of the bursa.

**Fig 3:**
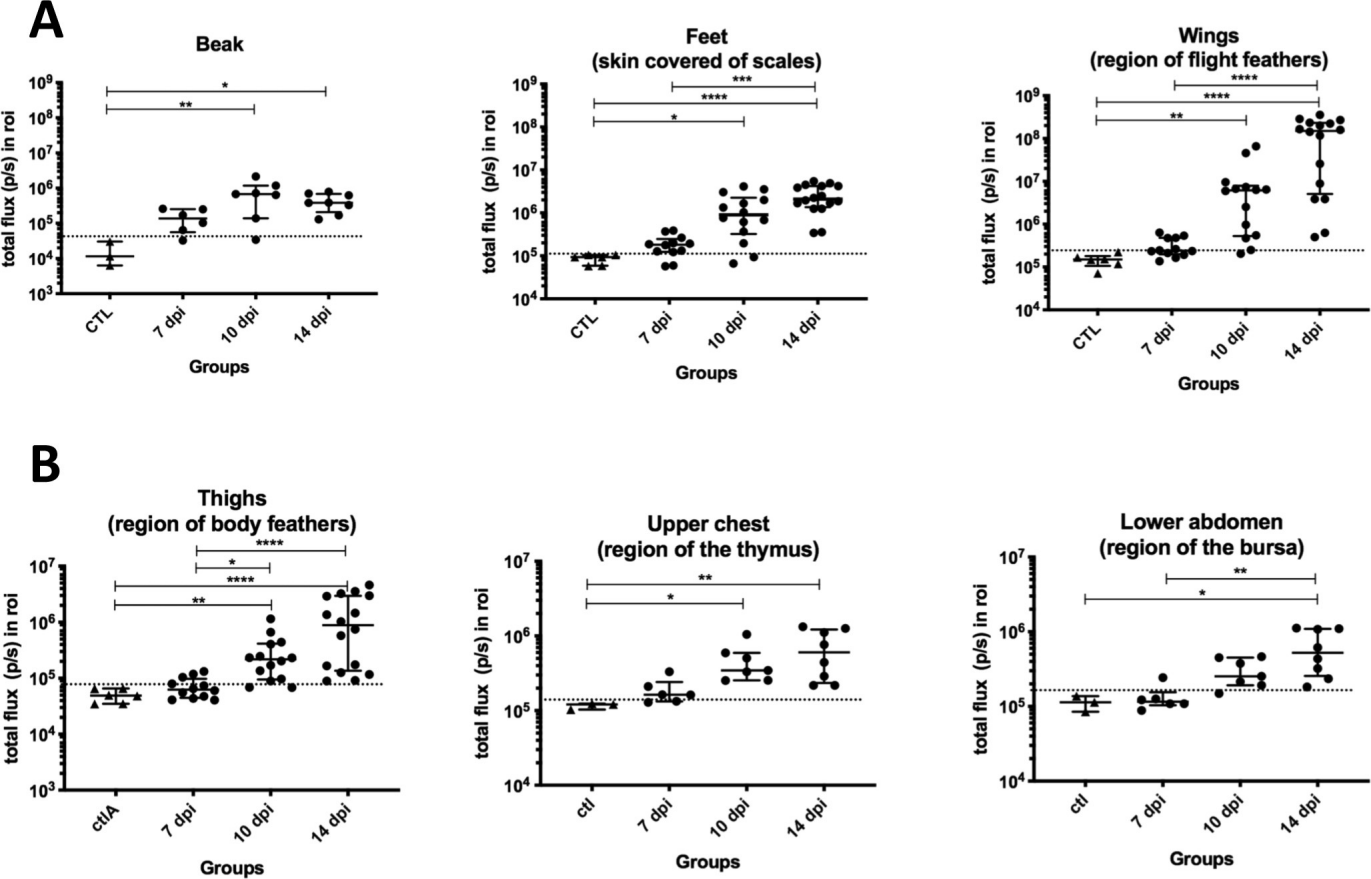
Bioluminescence *in vivo* imaging quantitation in vTK-fLuc-infected chickens. The bioluminescence signals were quantified per organ and group at each time point (7, 10 and 14 dpi) as defined (S2 Fig) and expressed in p/s (total flux). For this, regions of interest (ROI) corresponding to each organ/zone were defined. (A) Quantitation from five ROI corresponding to beak, feet (right and left) and wings (right and left). Each dot corresponds to one measure, the horizontal long bar to the median and the vertical bar to the interquartile range shown per group. For each graph, the threshold is indicated as a dotted line and was calculated as the mean of the three controls plus two standard deviations (beak, 4.16x10^4^ p/s; feet, 1.18x10^5^ p/s; wings, 2.47x10^5^ p/s). (B) Quantitation in four additional ROI: thighs (right and left), upper chest and lower abdomen. The symbols are the same as in (A). The thresholds were calculated for each ROI as earlier with the three controls (7.75x10^4^ p/s for the thighs, 1.38x10^5^ p/s for the upper chest, 1.65x10^5^ p/s for the lower abdomen). For (A) and (B), asterisks indicate significant differences (adjusted p-value <0.05, *; <0.01, **; <0.001, ***; <0.0001, ****; Kruskal-Wallis test with a Dunn correction for multiple comparison).

At 14 dpi, eight chickens (#1,4,6,10,11,12,13,14) were imaged a second time (Fig 2C). Despite variable intensities, all chickens presented a positive signal at the beak, feet and wings. At that time, as expected, the signal was intense at the basis of the wing feathers for most of the infected chickens. Four chickens (#4,6,12,14) displayed a weak signal at the tail feathers (rectrices). All traces of feces had been removed from the animals’ cloacae prior to imaging, indicating that this signal likely represents virus replication in the feather follicle epithelium of the tail feathers. To evaluate the changes of the bioluminescence signal over time, the signals were quantified in total flux in three anatomic regions: the beak, feet and wings (Fig 3A). Most of the beaks were luminescent from day 7. The total flux signal in the beak increased between 7 and 10 dpi and remained stable until day 14. The total flux in the beak differed significantly at 10 and 14 dpi compared to the control group (adjusted P value <0.01 and <0.05, respectively). In the feet and in the wing feathers, the total flux signal increased over time but less in the feet than in the wing feathers (Fig 3A). Similar to observations in the beak, the total flux differed significantly at 10 and 14 dpi in the feet and the wings compared to the control group (for the feet, adjusted P value <0.05 and <0.0001; for the wings, adjusted P value <0.01and <0.0001, at 10 and 14 dpi respectively). Intriguingly, luminescence signals appeared earlier in the beak and feet than at the basis of the wing feathers.

To evaluate the signal on feathered areas of the body other than the wings and tails, we defined an ROI on each thigh (S2 Fig) and quantified the signal in total flux (Fig 3B). Luminescence in the thighs had a pattern similar to that of the wings over time albeit weaker (Figs 3B and S2A). However, due to the very high signal in the wings for some birds at day 14 (*e*.*g*. chickens #11, #13, #14), we cannot exclude that the signal recorded in the thighs was partially or completely due to light emitted from the wings.

To determine if MDV infection was detectable in thymus and bursa of live animals, the total flux of luminescence was quantified in two additional ROIs also covered of feathers: the upper chest (for the thymus) and the bottom to the abdomen (for the bursa) (S2 Fig). At day 7, some birds showed values above the threshold, especially in the upper chest (#2,6) (Fig 3B). At 10 and 14 dpi, the signal in most chickens was positive although weak in both regions. The low intensity of the signals could be explained by feathers covering these areas blocking part of the signal or to the feathers themselves. Since our animal experimental license did not permit to pluck feathers from live animals, we examined the infection of these lymphoid organs post-mortem *in situ*.

### Infection kinetics in lymphoid organs and feathers follicles assessed by *ex vivo* bioluminescence

**Fig 4:**
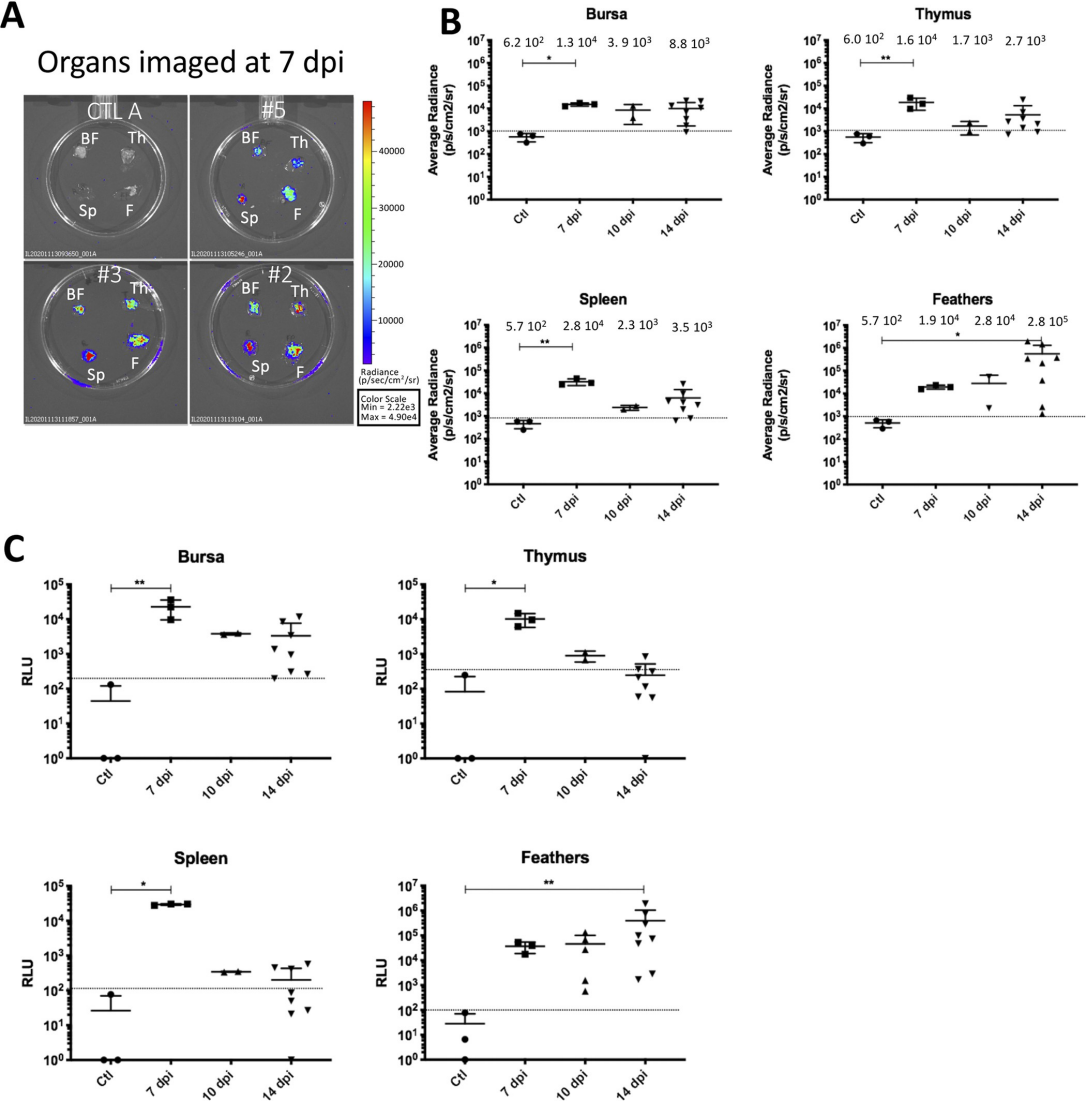
Bioluminescence imaging of organs from vTK-fLuc-infected chickens *ex vivo*. (A) Bursa (BF), thymus (Th), spleen (Sp) and feathers material (F) of three infected chickens (#2, #3 and #5) imaged at 7 dpi by IVIS Spectrum. All tissues were bioluminescent at 7 dpi indicating infection. The organs of a non-infected control chicken matched for age are shown for comparison. (B) Bioluminescence measurements by organ imaged with IVIS Spectrum at each time point. The bioluminescence is expressed in average radiance (p/s/cm2/sr). For each time, the mean is shown as a black line (value indicated above) with standard deviations. All lymphoid tissues showed a significant increase in bioluminescence at 7 dpi indicating lytic infection and the feathers were significantly luminescent at 14 dpi. Asterisks indicate significant differences (adjusted p-value <0.05, *; <0.01, **; <0.001, ***; <0.0001, ****; Kruskal-Wallis test with a Dunn correction for multiple comparison). (C) Bioluminescence measurements by organ and time points performed from crude organ extracts with an *in vitro* luciferase assay. The dashed horizontal lines (B and C) indicate the threshold (the limit of positivity) of detection of the bioluminescence signal, set as the mean of the three negative controls plus 2 standard deviations. All lymphoid tissues were significantly bioluminescent at 7 dpi indicating lytic infection and the feathers were significantly fluorescent at 14 dpi. Asterisks indicate significant differences (adjusted p-value <0.05, *; <0.01, **; <0.001, ***; <0.0001, ****; Kruskal-Wallis test with a Dunn correction for multiple comparison).

To assess the infection of the bursa, thymus and spleen, all vTK-fLuc-infected chickens from the previous experiment were sacrificed at 7, 10 or 14 dpi. Immediately after euthanasia, organs were harvested, a piece of each organ was prepared, finely chopped and then covered with D-Luc and imaged in IVIS Spectrum. In addition, feather tips from the wing feathers, containing the feather pulp and epithelium, were collected and examined along with the lymphoid organs. Organs from a non-infected bird were imaged at the same time as a negative control to determine the threshold. For all tissues from infected birds, a luminescence signal was clearly detectable at 7 dpi (Fig 4A). The average radiance in all assessed tissues was highest at day 7 (with a median of above 10^4^ p/s/cm^2^/sr), except for the feathers (Fig 4B). The median average radiance in the bursa, thymus and spleen differed significantly at 7 dpi (adjusted P value <0.05, <0.01 and <0.01 respectively) compared to the control group. The signal in thymus and spleen decreased slightly over time, while it remained stable in the bursa until 14 dpi. At these time points, the median average radiance in lymphoid organs did not differ significantly compared to control animals. In case of the feather tips, the bioluminescence signal continuously increased until day 14 (with median of 1.9 10^4^ at day 7 to 2.8 10^5^ p/s/cm^2^/sr at day 14). The median average radiance in this tissue differed significantly at 14 dpi (adjusted P value <0.05) compared to the control group. To further validate our data, we also measured the luciferase activity using a conventional luminometer from lysed tissues (lymphoid organs, feather tips) (Fig 4C). The dynamics of the bioluminescence signal measured in relative luminescence (RLU) was similar to IVIS Spectrum in terms of kinetics and differences between groups; however, this method appeared to be slightly less sensitive than the IVIS Spectrum.

### vTK-Luc replication in the skin of the feet

**Fig 5:**
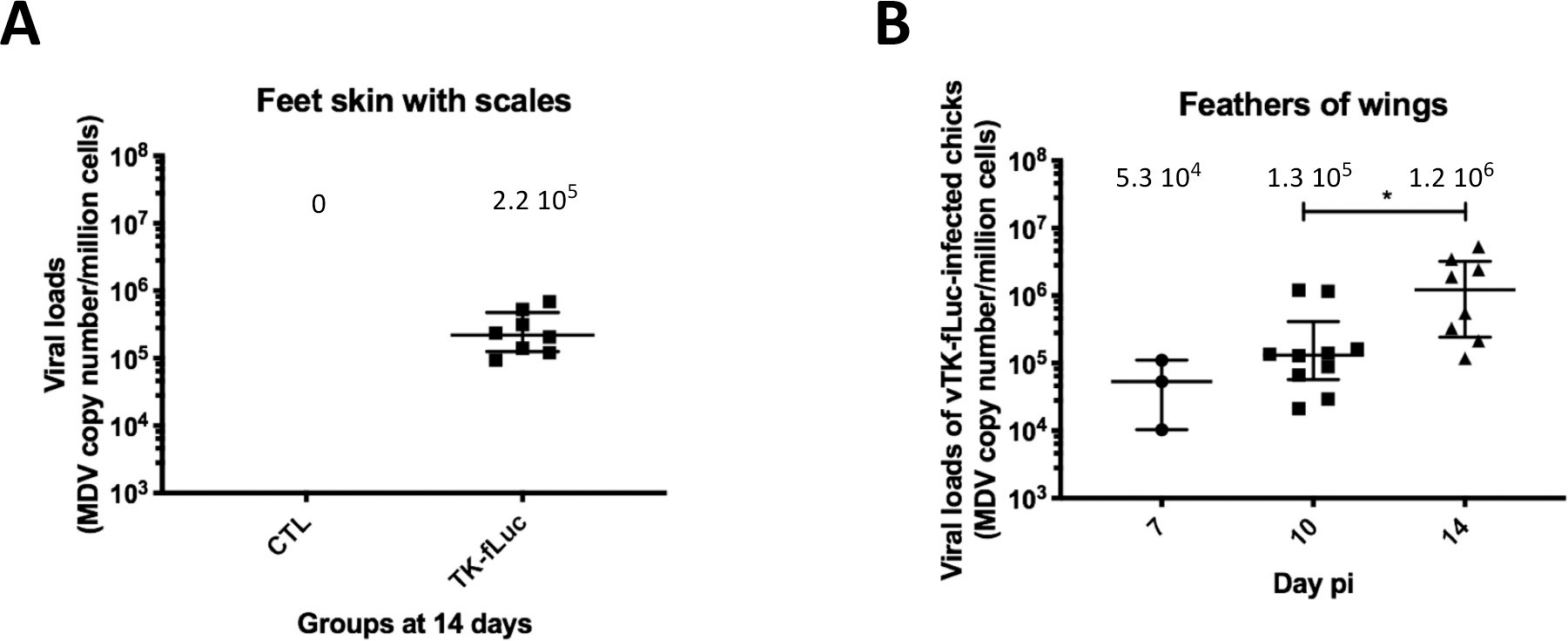
Viral genome loads in the feet skin of vTK-fLuc-infected chickens. Viral genomes were quantified (A) in feet skin of chickens at 14 dpi as well as (B) in wing feathers tips material at all time points (positive control) by real-time qPCR and their numbers indicated per million cells. For each group, the median is shown as a black line (value indicated above) with the interquartile range. In (A), feet skin samples from three 14-day old control chickens were analyzed. In (B), the asterisk indicates significant differences (adjusted p-value <0.05, *; Kruskal-Wallis test with a Dunn correction for multiple comparison).

To determine whether the bioluminescence signal at the feet skin level reflects virus replication, we quantified the presence of viral DNA in this tissue. For this, the dorsal side of foot skin (metatarsus and toes) covered in scales of all birds necropsied at 14 dpi was dissected. DNA was extracted and viral loads measured by qPCR (Fig 5A). Wing feathers at all time points were used as a positive control (Fig 5B). It should be highlighted that an explicit positive correlation between in vivo bioluminescence signals and viral DNA loads was observed in this tissue (S3 Fig). Though slightly lower than in feathers measured at the same time (median of 1.2x10^6^ genome copies/million cells), high levels of MDV genomes (median of 2.2x10^5^ genome copies/million cells) were detected in all eight skin feet samples at 14 dpi, highlighting that the virus efficiently infects the skin of the feet. No viral genome was detected in the feet skin of control chickens at 14 dpi.

### Validation of lytic infection of the feet skin using a fluorescently-labeled virus

**Fig 6:**
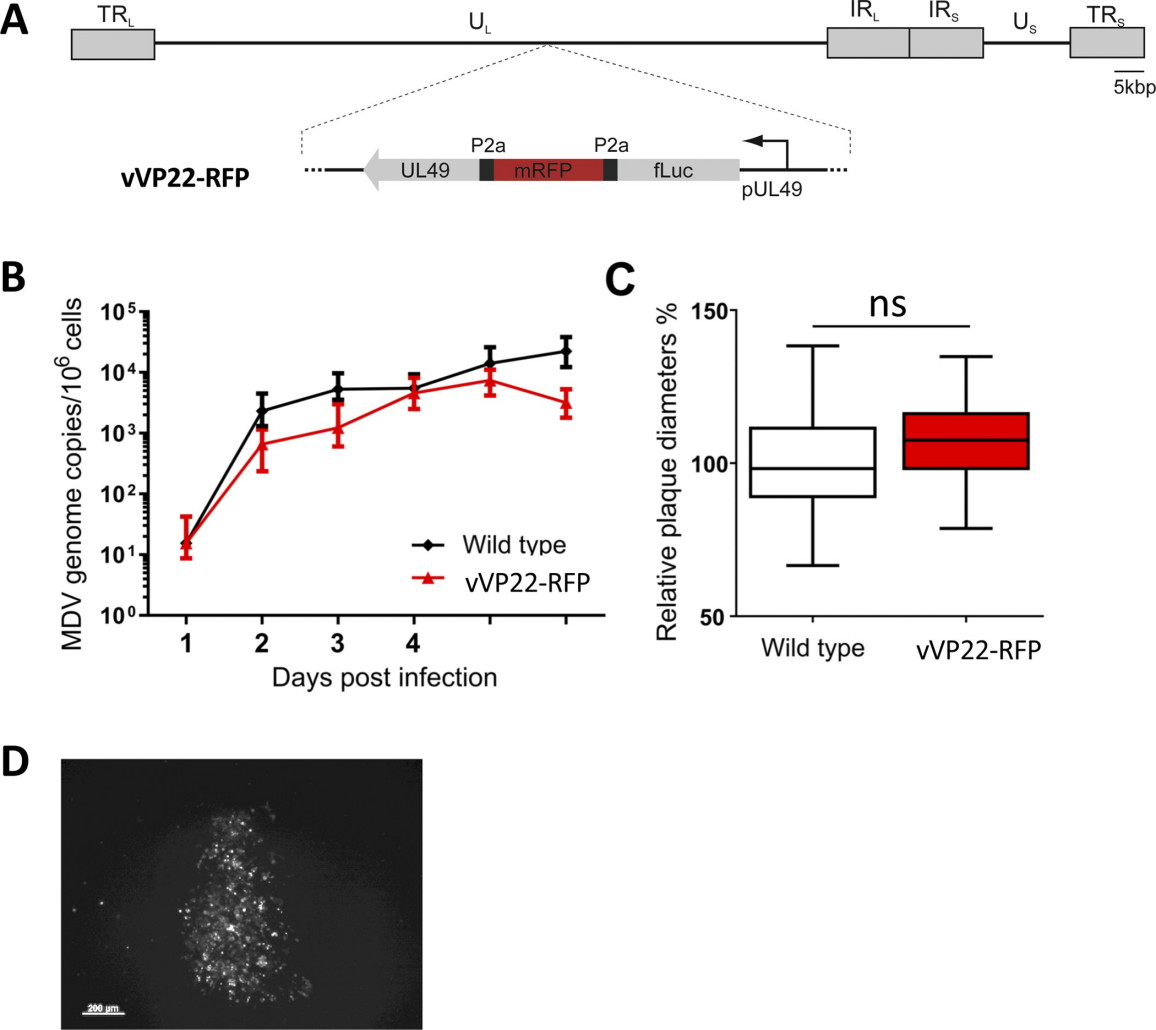
vVP22-RFP reporter virus replication in culture. (A) Schematic representation of the vVP22-RFP genome. This RB-1B mutant harbors fLuc-P2a-mRFP1-P2a inserted at the 5’ end of the UL49 gene encoding VP22, a major tegument protein highly expressed during lytic replication. Replication was assessed by (B) multi-step growth kinetics 1 to 6 dpi and virus spread by (C) plaque size assay (n = 150). Spread and replication of indicated viruses were not statistically different (p > 0.05, one-way ANOVA). (D) Image of a vVP22-RFP plaque taken by fluorescent microscopy, revealing a moderate fluorescent signal in culture.

To determine if the bioluminescence signal detected with vTK-fLuc in the wing feathers and feet skin stemmed from lytic MDV replication, we generated a second recombinant virus using the bacterial artificial chromosome (BAC) system of the very virulent RB-1B strain that expresses monomeric Red Fluorescent Protein (mRFP) during the late phase of the lytic cycle (vVP22-RFP). The mRFP gene was inserted at the 5’ end of the UL49 gene encoding the major tegument VP22 protein. To optimize its expression and minimize potential spurious effects of a reporter gene fusion on VP22 function, mRFP as well as fLuc were linked using P2A self-cleaving peptides (Fig 6A), resulting in the expression as three separate proteins (mRFP, fLuc and VP22) with comparable kinetics. Upon reconstitution, multi-step growth kinetics and plaque assays revealed that VP22-RFP replicates comparable to the parental rRB-1B virus (Fig 6B and 6C). The fLuc signal was hardly detectable, and therefore not used further. In contrast, the mRFP signal was readily detectable by fluorescence microscopy (Fig 6D).

**Fig 7:**
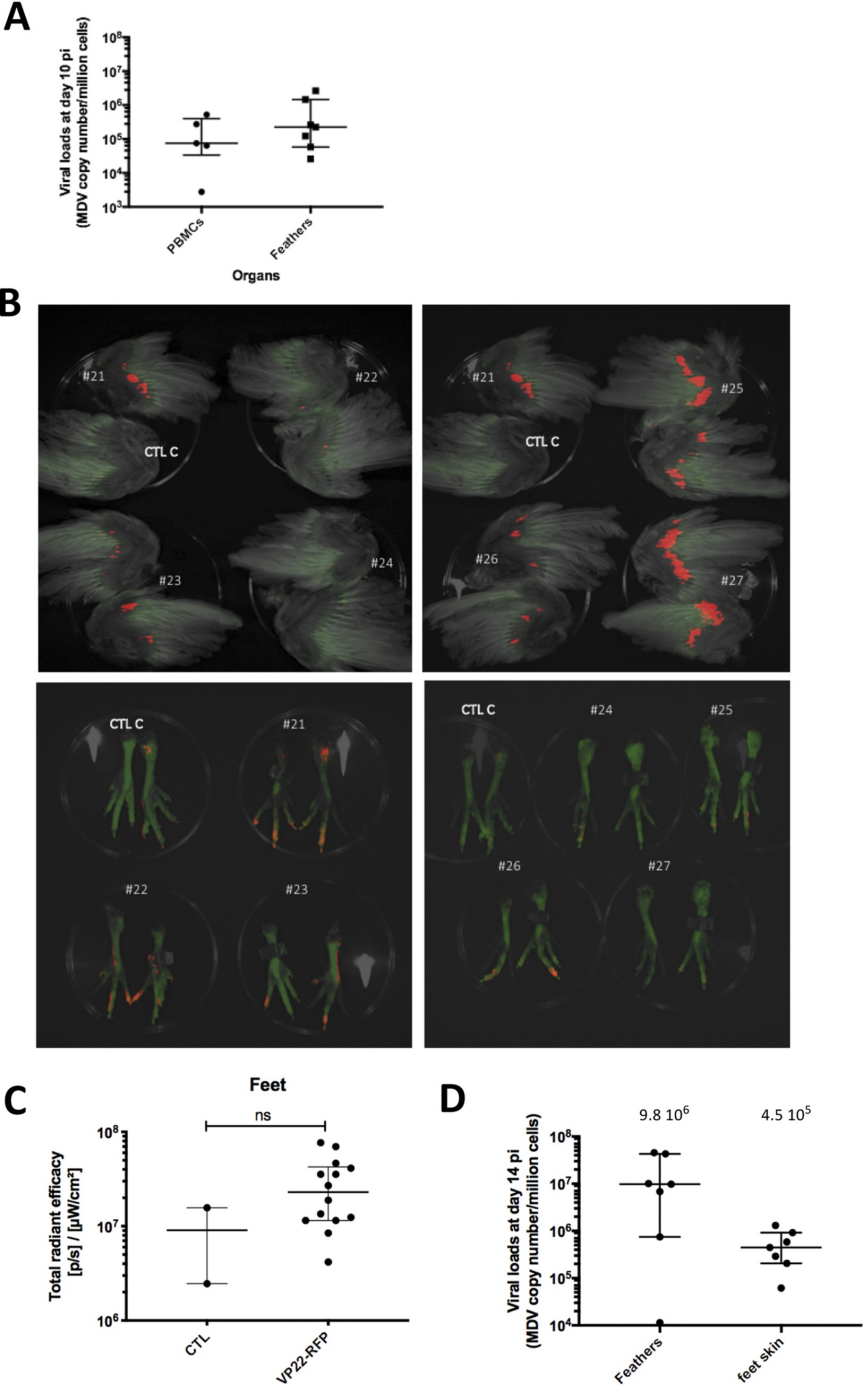
Fluorescent *ex vivo* imaging of vVP22-RFP-infected chicks. (A) Viral genome loads measured at 10 dpi in PBMCs and feather tips material. (B) Wings and feet of euthanized chicks were imaged at 14 dpi with an IVIS Spectrum. The red fluorescence was acquired using the spectral unmixing mode. Wings and feet from a control chicken matched for age (CTL C) were imaged for comparison. C. Quantification of the RFP signal on the feet. The quantification was performed using the IVIS software on each foot taken as one ROI. Each dot corresponds to one measure, the horizontal long bar to the median and vertical bar the interquartile range shown per group. Though some animals present a relatively high signal, the difference of fluorescence between the VP22-RFP infected chickens and the control was not significant (Mann-Whitney test, exact p-value = 0.2583, ns. D. Viral genome loads measured at 14 dpi in feather tips material and skin of the feet. For each group, the median is shown as a black line (value indicated above) with the interquartile range.

One-day-old SPF WL B13 chickens (n = 7) were next infected intramuscularly with 2000 pfu of vVP22-RFP. Infection of the chickens was confirmed by qPCR on PBMCs and feathers collected at 10 dpi (Fig 7A). Fluorescence imaging was performed in the spectral mode. Since this imaging mode takes longer than the permitted duration of anesthesia, we euthanized the birds at 14 dpi and imaged the limbs post-mortem. The red fluorescent signal was clearly detectable at the basis of the wings in most of infected chickens (Fig 7B), with varying intensity as described earlier for the bioluminescence. Importantly, we also confirmed the lytic infection of the feet using the fluorescently labeled virus, which was mostly localized in the toes (Fig 7B). The intensity of the mRFP signal varied between the wings and feet of individual animals. For example, chick#22 had a very faint signal in the wings and one of the strongest in the feet. Conversely, chick#27 showed a strong signal in the wings and almost no signal in the feet. Despite a clear and marked signal in the feet of three chickens (#21, 22, 26), a quantification of the signal in total radiant efficiency ([p/s]/[μW/cm^2^]) from the feet of all infected chickens revealed no significant differences between the infected animals compared to control birds (Fig 7C). To corroborate the fluorescence imaging data, viral loads in the wing feathers and feet were measured by qPCR (Fig 7D). Like with the vTK-fLuc, the viral genome copies of the infected birds matched the fluorescent signal intensities very well, confirming that wing feathers and skin of the feet are indeed infected.

### Detection of infectious virus in feet skin keratinocytes using a TK-GFP virus

**Fig 8:**
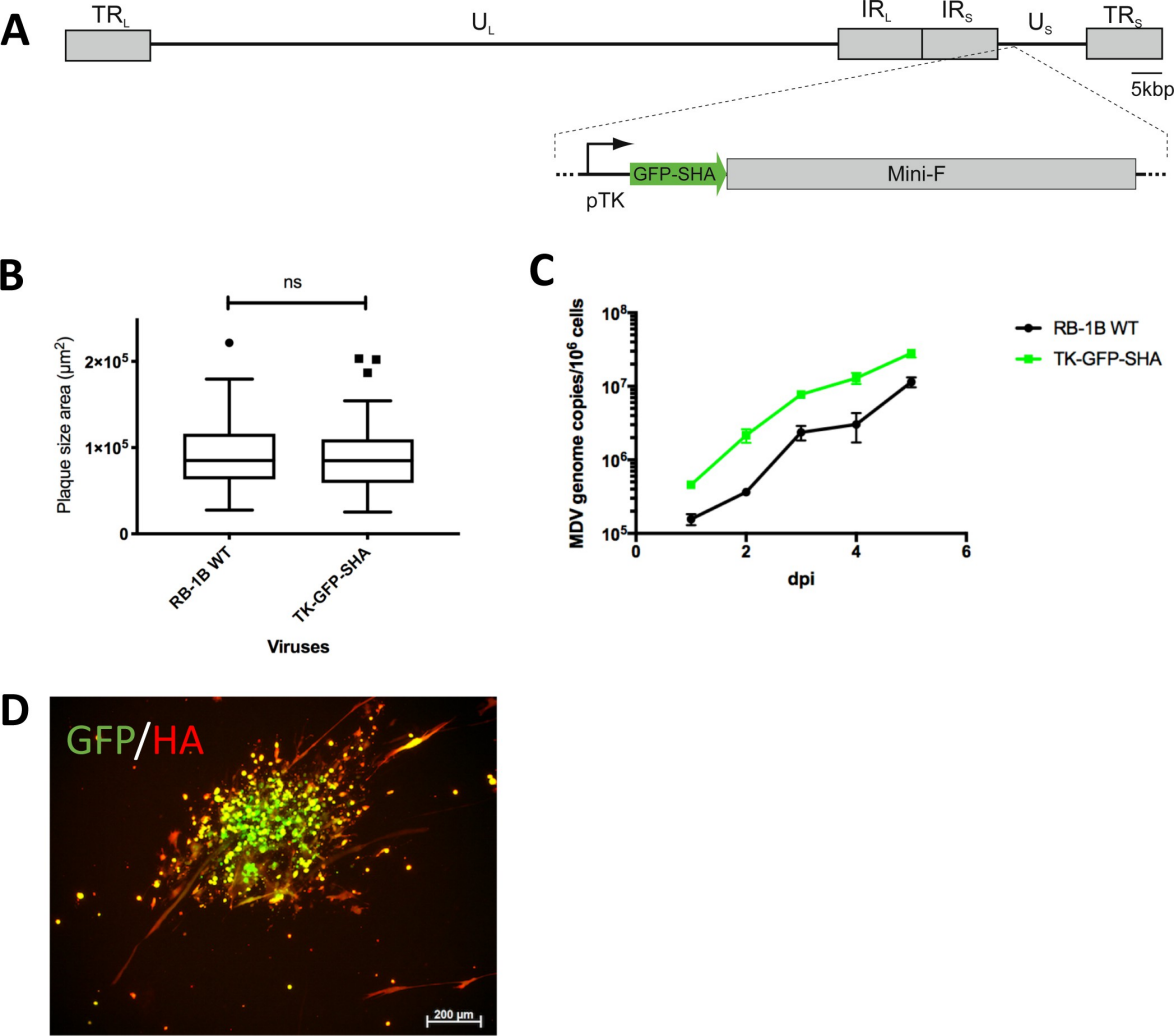
vTK-GFP-SHA reporter virus replication in culture. (A) Overview of the MDV genome containing the vTK-GFP-SHA cassette. (B) vTK-GFP-SHA spread in cell culture was assessed by a plaque size assay (n = 70) compared to the parental WT rRB-1B virus. (C) Replication was assessed by multi-step growth kinetics 1 to 5 dpi. (D) Image of a TK-GFP-SHA plaque taken by fluorescent microscopy, after immunostaining with an anti-HA antibody plus a goat anti-mouse-Alexa Fluor 555, revealing a high GFP fluorescent signal and an expression of the HA tag in culture.

**Fig 9:**
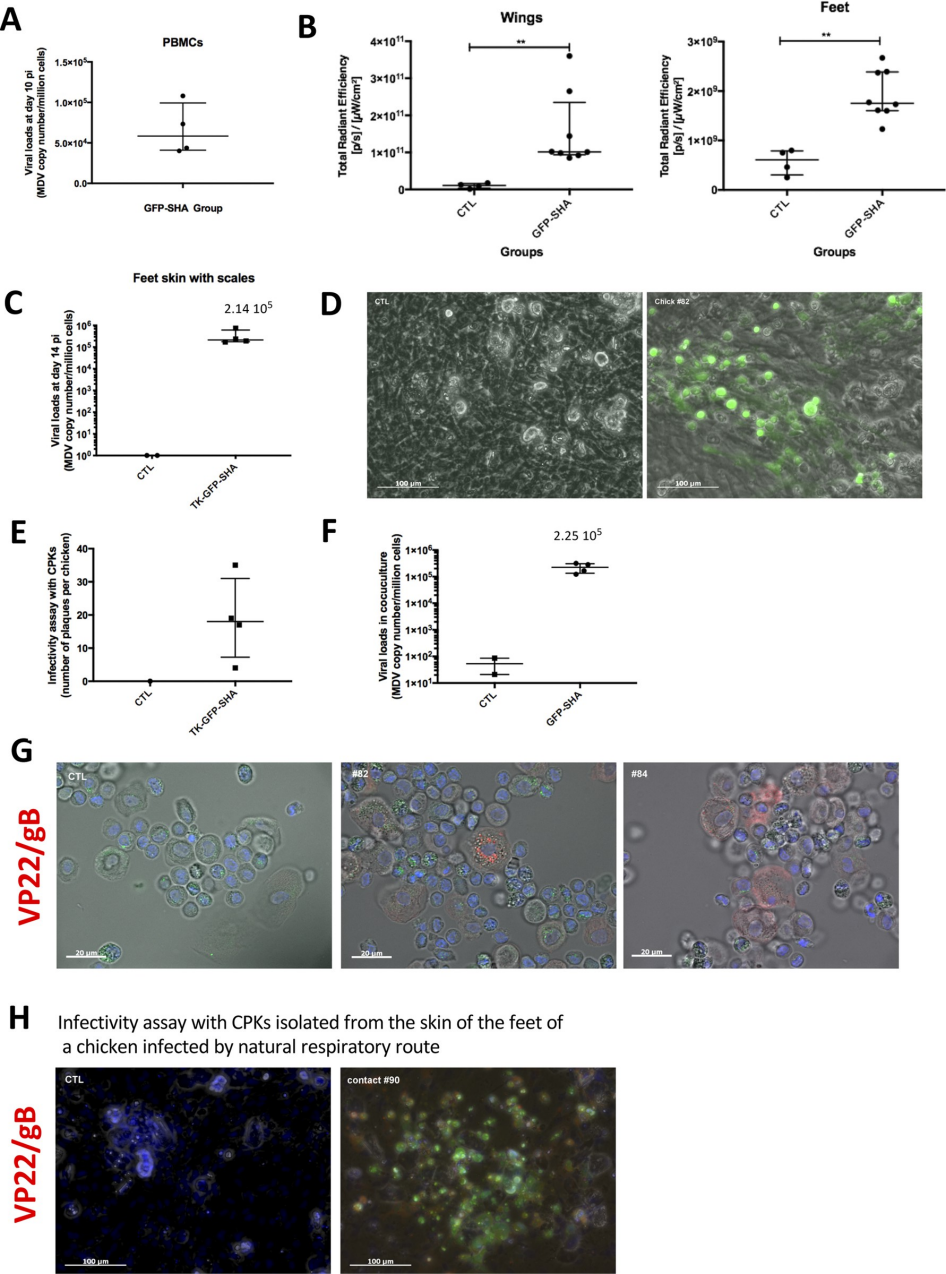
Fluorescent *ex vivo* imaging with the vTK-GFP-SHA and infectivity from the skin of the feet. (A) Viral genome loads measured at 10 dpi in PBMCs. The median is shown as a black line with the interquartile range. (B) Quantification of the GFP signal on the wings and the feet. The quantification was performed using the IVIS Spectrum software on each wing and foot taken as one ROI. Each dot corresponds to one measure, the horizontal long bar corresponds to the median and the vertical bar to the interquartile range per group. (C) Viral loads measured at 14 dpi in the skin of the feet. The median is shown as a black line with the interquartile range. Infectivity assessed by a plaque assay from four infected-chickens (14 dpi) and a control (D, E, F). Primary keratinocytes isolated from the skin of the feet was co-cultivated with CESCs for 4 days. (D) Pictures of an infection plaque (chick #82) and of a non-infected layer (control chick). The GFP was directly detected in the green channel. (E) Number of plaques obtained by coculture for each chicken at 14dpi. (F) Viral loads measured in the coculture of the infectivity test. Each dot corresponds to one chicken, the horizontal long bar to the median and the vertical bar to the interquartile range per group. (G) Primary keratinocytes isolated from the skin feet of infected-chicken, 14 days post-injection. Keratinocytes were stained with mouse monoclonal antibodies to MDV proteins (gB and VP22), revealed with a secondary antibody coupled to Alexa Fluor 594 (red). The nuclei were stained with Hoechst 33342 (blue). The cell autofluorescence and the GFP are visible in the green channel. (H) Infectivity assessed by a plaque assay from a chicken infected by natural route; a control chicken is also shown. The cocultures were stained with mouse monoclonal antibodies to MDV proteins (gB and VP22), revealed with a secondary antibody couplet to Alexa Fluor 594. The nuclei were stained with Hoechst 33342 (blue). The GFP was directly detected in the green channel.

To corroborate the previous findings and to determine if the *in vivo* luminescence and fluorescence signals observed in the feet skin would reflect MDV shedding, we generated a third RB-1B recombinant, vTK-GFP-SHA, using the bacterial artificial chromosome (BAC) system of the very virulent RB-1B strain. This mutant expresses the Green Fluorescent Protein (GFP) fused with an HA-tag and a strep-tag under the control of the early HSV-1 TK promoter (Fig 8A). Plaque size assay and multi-step growth kinetics showed that vTK-GFP-SHA spreads and replicates in culture *in vitro* comparable to the parental rRB-1B virus (Fig 8B and 8C). This virus allows detection of the virus directly through GFP fluorescence or indirectly through immunofluorescence staining targeting GFP or the HA tag (Fig 8D). In an additional animal experiment, six five-day-old WL B13 SPF chickens were infected intramuscularly with 3000 pfu of the vTK-GFP-SHA and housed with four non-infected age-matched chickens (contacts). Infection of the chickens was confirmed by qPCR on PBMCs collected at 10 dpi (Fig 9A). Four inoculated birds were euthanized at 14 dpi for post-mortem bioimaging of the limbs in the IVIS Spectrum using the fluorescence mode. The GFP fluorescence signal was clearly detectable in the spectral mode at the basis of the wings and in the feet of all chickens (Figs 9B and S4A). The median total radiant efficiency in the wings and the feet differed significantly at 14 dpi (exact p value <0.01 for both limbs) compared to the control animals (two age-matched uninfected birds). The presence of MDV in the feet skin was confirmed by qPCR (Fig 9C). We next explored the presence of infectious viruses in the feet skin. Because in feather follicles replication is known to occur only in epithelial cells, we focused our analyses on keratinocytes in the feet skin. Primary keratinocytes (CPKs) from one foot skin sample per chicken were isolated and a third of this cell preparation was co-cultivated with CESCs for 4 days. GFP-positive virus plaques were visible upon CESC co-cultivation with CPKs from the four infected chickens (Fig 9D and 9E). No signs of infection were seen upon CESC co-cultivation with CPKs from the uninfected control birds (Fig 9D and 9E). Viral loads in the infected co-cultures were quantified by qPCR (Fig 9F). Finally, we assessed the expression of late lytic MDV antigens in isolated CPKs. To this end, isolated CPKs were immuno-stained with a cocktail of two primary monoclonal antibodies targeting the glycoprotein B and the major tegument protein VP22 (Fig 9G).

The remaining two inoculated birds in this experiment were euthanized at 35 dpi and showed tumors, indicating that TK-GFP-SHA is oncogenic and not attenuated. Primary keratinocytes (CPKs) from a foot of these two infected chickens and of a control chicken age-matched were isolated and co-cultivated with CESCs as earlier. GFP viral plaques were visible with the CPKs of both infected chickens at 35 dpi (5 plaques with chick#83, 6 with chick #85) and not of the control. Interestingly, most beaks (inferior and superior) and claws also showed small GFP foci when observed under a fluorescent stereomicroscope (S4C Fig). In the beak, the signal is mostly at the extremity. In addition, we also observed clear signal and infectious virus in the foot skin of a contact chicken that was infected via the natural route of infection (Fig 9H).

### Localization of the lytic infection in the feet skin epithelium

**Fig 10:**
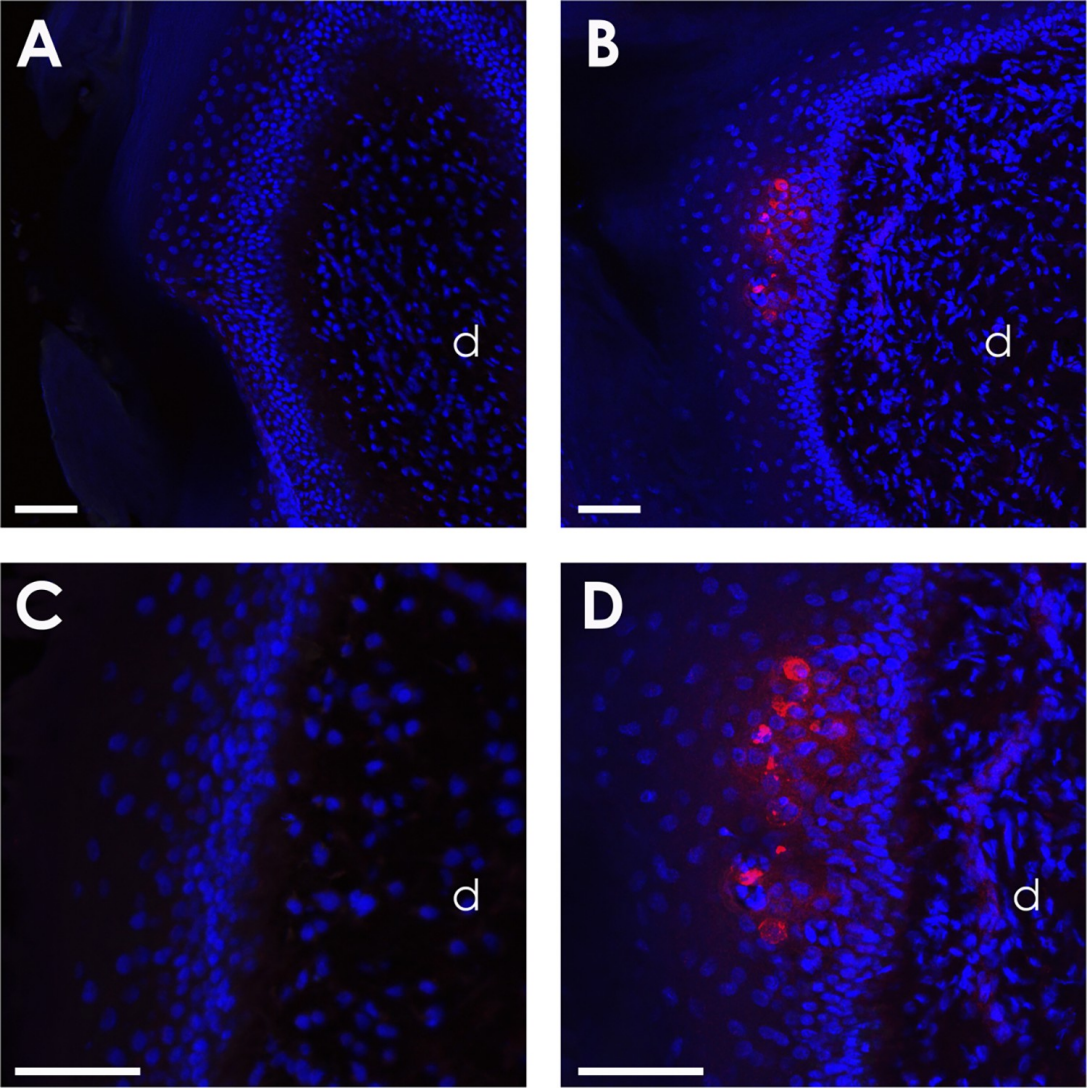
Localization of GFP in the feet skin from vTK-GFP-SHA-infected chickens at 14 dpi. Control animal (Left panel). MDV-infected animal (Right panel). In MDV-infected animals only, clusters of acanthocytes with intracytoplasmic signal (red) were detected in the epidermis above the basal layer. No GFP-associated signal was observed in dermis (d). Indirect immunofluorescent detection with a polyclonal anti-GFP antibody and DAPI nuclear counter-staining. The scale bar corresponds to 50 μm.

To determine in which layers of the foot epidermis the MDV infection was located, the feet skin areas with the highest fluorescence signal were dissected from the TK-GFP-SHA inoculated chickens at 14 dpi and frozen. Cryo-sections were stained with an anti-GFP antibody and a secondary antibody coupled to a red fluorochrome and observed under a confocal microscope. Infection foci were detected in which the infected cells were keratinocytes of intermediate epidermal layers (Fig 10). As expected, the GFP staining signal was located in the cytoplasm. Some infected cells showed fragmented nuclei suggesting a cytopathic effect induced by lytic viral infection. It should be noted that none of the keratinocytes in the basal epidermal layer and the cells of the dermis were GFP-positive.

## Discussion

It is well established that MDV transiently replicates in lymphoid organs early during the viral life cycle from 4–10 dpi [11, 21]. Replication in the feather follicles occurs later from 8–10 dpi until the death of the animal [17, 22, 23]. In this study, we confirmed the early replication of MDV *in vivo and ex vivo* using bioluminescent imaging in chickens for the first time. Using this approach, we discovered two novel sites of virus replication in hard skin appendages: the feet skin covered with scales and the beak.

Bioluminescence signals were readily detected in infected chickens at the basis of the wing feathers (including feather follicles and outer feather sheaths). The signals were detected in almost all chickens from day 7 and in all animals at 14 dpi, with an increase of the signal intensity over time. We therefore detected reporter protein expression two days earlier than previously reported. Indeed, Jarosinski reported MDV antigen expression in the feather follicle epithelium only from 9 dpi (and in less than 10% of feather follicles) using a recombinant virus harboring mRFP fused to the UL47 gene and a microscopy approach [22]. This difference could be due to the late expression kinetics of UL47 compared to TK-fLuc, different intensities of the reporter genes or different sensitivity of the imaging techniques. In our study, all chickens had a clear luminescence signal at the wings at 10 dpi, except chicken #10 that also had a much lower viral load of 2.94 10^4^ copies/10^6^ cells in the feathers, a result showing the relationship between the bioluminescence and the viral load. This chicken was clearly positive at day 14, indicating a delay in the spread to the feathers. In addition to the wing feathers, a bioluminescence signal was detected in the tail feathers of four chickens at 14 days post-infection. Interestingly, wings and tail feathers are the largest feathers present at that early age and the first juvenile feathers replacing down (teleoptile feathers) appearing on young chickens. The wings’ flight feathers appear already *in ovo* before hatch [24], whereas the tail feathers appear after hatch, in the first week of age, and the body feathers not before 18 days of age for the WL B13 birds used in this study. It is intriguing that the bioluminescence signals were mostly detected in these rapidly growing juvenile feathers and not in the down feathers, representing already cornified feathers. This suggests that MDV preferentially enters and replicates in growing feather follicles, which clearly merits further investigation. Such knowledge is important to understand how feathers get infected with MDV in order to develop new vaccines that are able to block virus shedding and dissemination.

One exciting aspect of our study is the discovery of MDV infection and replication in the feet skin, on the anterior metatarsus and the dorsal part of the toes that are covered with scales. This was observed with bioluminescence of vTK-fLuc at all time points and fluorescence of vVP22-RFP at 14 dpi. Surprisingly, at 7 dpi, more birds infected with vTK-fLuc exhibited a signal in the feet than in the feather follicles of the wings, suggesting that replication starts earlier in the feet than in the feather follicles. The intensity of the luminescence signal increased in the feet over time, even though it remained at lower levels compared to the feather follicles. The presence of MDV genome in the skin of the feet of all infected chickens (both viruses) analyzed at day 14 confirmed this interesting finding. This was further corroborated by using an in vivo bioimaging approach with two fluorescent viruses, vVP22-RFP and vTK-GFP-SHA, which could also be detected in the feet. The fluorescence signal was faint but significant in many infected birds, notably with the vTK-GFP-SHA. The high brightness of the vTK-GFP-SHA may explain that this virus was better detected in fluorescence in the feet than the vVP22-RFP. MDV replication in the feet skin was confirmed by immunohistochemistry, revealing small foci of viral infection, an observation that may explain the faint signal. Moreover, MDV replication was only observed in the intermediate layers of the feet skin epidermis and not in the basal layer, exactly as previously reported for the feather follicle epithelium [18]. The presence of replicating virus only in intermediate layers in the feather follicle and the feet skin epithelium indicates that keratinocyte differentiation could play an important role in MDV replication as previously described for papillomaviruses [25]. In addition, primary keratinocytes isolated from the feet epidermis of infected birds were found to readily transmit MDV infection regardless of the route of animal infection (intramuscular inoculation or direct contact transmission). Finally, this was demonstrated for birds infected at 5-day-old at different times post-infection (14 and 35 dpi), indicating that productive infection of the feet skin is not a transient phenomenon but persistent for at least three weeks post-infection. The regions of the skin of the feet that showed luminescent or fluorescent signals are the only ones covered in scutate scales [26]. Such scales have (i) an outer surface composed of corneous beta-proteins (previously named ß-keratins) like other hard integuments (feathers, beak and claws) and (ii) an inner surface composed of alpha-keratins like the epidermis of the skin without feathers (inter-appendage or nude regions) [26–28]. Interestingly, it has been hypothesized that scutate scales have secondarily evolved from feathers and are not homologous of scales from reptiles [29, 30]. It is intriguing that MDV replicates in hard integumentary structures and not in the skin epidermis in general. Further studies on these aspects in this particular epidermis could provide important insights into the molecular determinants of the tropism and virion production of MDV. In addition, to date, the feather follicles were the only tissue known to release fully infectious MDV virions into the environment [16], resulting in a high viral load in poultry dust. We hypothesize that the scutate scales could also shed infectious virus and be another source of MDV horizontal transmission.

Luminescence or fluorescence signals were also detected in the beak with vTK-fLuc virus and the TK-GFP-SHA virus respectively. As we had difficulties to dissect the beak and isolate living cells, we could not confirm the presence of infectious MDV nor locate the potential replication sites in this tissue. Contaminated dust shed from feathers or feet skin is likely not the source of the fLuc signal in the beak, because only living cells that received D-luciferin via systemic spread within 10 min can emit luminescence. This was validated with the fluorescent signal of TK-GFP-SHA infected chickens, at 35 dpi. The surface of the beak being completely cornified and composed of dead cells, we hypothesize that the luminescence signal most likely comes from the growth zone of the beak [31].

We also observed faint bioluminescence signals in the lymphoid organs through post-mortem *in situ* imaging, from 7 to 14 dpi, in all bursa examined as well as in most of the thymus or spleen samples. The low intensity of the signals is consistent with the fact that only a small percentage of cells in the lymphoid organs are infected as described previously [21, 32, 33]. The persistence of a bioluminescence signal until 14 dpi in the three lymphoid organs of most animals was not expected, because we and others previously reported that MDV mostly replicates in these organs between 3 and 7 dpi and subsequently establishes latency [11, 21, 33, 34]. Nevertheless, in a previous infection study with the RB-1B strain, we had also observed sparse and rare MDV-positive cells at day 14 post-infection particularly in the bursa [33]. To explain these late signals herein, the most plausible assumption is that bioluminescent imaging technique is more sensitive than other techniques used earlier such as microscopy and flow cytometry or that this a particular feature of RB-1B infection. The superior sensitivity of IVIS Spectrum analysis could be attributed to the high sensitivity of the bioluminescence itself, but also to the sampling of a large piece of the fresh organ (unlike for microscopy), directly analyzed without any pre-treatments during which infected-cells may be lost (unlike for cytometry). Altogether, our data suggests that MDV replicates at least to some degree until 14 dpi in the lymphoid organs, notably in the bursa. In contrast to *ex vivo* imaging, we only detected minimal signals *in vivo* in the areas of the body containing the lymphoid organs. This was not totally surprising and could result from a conjunction of several factors: (i) the weak signals recorded in the *in-situ* imaging in these organs; (ii) the well-known physical limits of detection of fLuc luminescent signal. Indeed, the emitted light is blocked by tissues and is only detectable from a low depth of the skin covering the body surface (probably less than 1 cm) [3, 35]. This phenomenon may be stronger due to the presence of down and the sized of the chicks that are much larger than an adult mouse.

Finally, this study confirms that IVIS *in vivo* imaging system is particularly well suitable to track skin infections. This method revealed for the first time the infection of three different integument structures in young chickens. It also allowed us to examine all surface of this relatively large animal compared to other vertebrate models previously used for *in vivo* imaging, such as mice or zebra fish. Still, due to the very rapid growth of chickens after hatch, imaging of entire chickens older than 2–3 weeks is likely more challenging if not impossible. For MDV infection, this approach provided important new insights, especially regarding rapid spread of the virus to the hard skin appendages.

In summary, we established *in vivo* imaging of an infection in live chickens and we were able to track a viral pathogen in these animals for the first time. Using this method, we identified two novel sites of MDV replication never suspected before, the beak and the skin of the feet, underlining the importance of whole-body imaging. We also demonstrated that the virus replicates in epidermis of the feet skin and is fully infectious. In future studies, we will assess if these replication sites contribute to virus shedding into the environment and transmission within a population. Overall, our study demonstrates that bioluminescent imaging represents an exciting tool to assess the tropism of avian pathogens (incl. viruses, bacteria and parasites), especially to the skin of chickens and other bird species.

## Materials and methods

### Ethics statement

*In vivo* experiments were carried out according to the guidance and regulation of the French Ministery of Higher Education, Research and Innovation (MESRI) with well-trained staff, good animal practices and project authorizations (Protocol Number APAFIS19150-2019021315488293 v6). As an integral part of this process, the experimental protocol was examined and approved by the appropriate local ethics committee, CREEA VdL (“Comité d’Ethique pour l’Expérimentation Animale Val de Loire).

### Cells

Chicken embryonic cells (CECs) were prepared from VALO SPF 11-day-old embryonated chicken eggs (Valo BioMedia; Osterholz-Scharmbeck, Germany) as described previously [36]. Chicken embryonic skin cells (CESCs) were prepared from WL B19 SPF 12-day-old embryonated chicken eggs (INRAe) and cultivated as described previously [37].

### Generation of MDV reporter viruses

Luciferase reporter or fluorescently labeled viruses were generated using a bacterial artificial chromosome (BAC) of the very virulent RB-1B strain (kind gift of Dr V. Nair) [38] using the two-step Red-mediated recombination system as described previously [39, 40]. To generate the luciferase reporter virus, we first generated a luciferase transfer construct (epPCRfLuc) based on the psiCHECK2 vector (Promega) and fLuc gene, which was codon optimized for human cell expression. The kana_I-SceI cassette was amplified from the pEPkan-S plasmid [39] using primers (see Table 1) containing homologue sites for its removal, and cloned into the *NdeI* site of the psiCHECK2 vector. The luciferase mutagenesis cassette was subsequently amplified by PCR using primers (see Table 1) containing homologue sites for the insertion into the mini-F in place of the xGPT gene behind the HSV-1 thymidine kinase (TK) promoter, resulting in the recombinant vTK-fLuc. vTK_GFP-SHA was constructed on the backbone of vTK_GFP [41]. It encodes GFP with a 3’end in-frame fusion of a HA-/Strep-tag (SHA) driven by the thymidine kinase promoter. To generate the vTK_GFP-SHA, the SHA mutagenesis cassette was first amplified by PCR from the SHA template plasmid (pKanInSHA), synthetized by GeneArts, using primers containing the appropriate end sequences (Table 1). This PCR fragment was next used for the insertion of SHA at the 3’end of GFP by red-recombination into the BAC RB1B TK-GFP. In addition, we generated a RB-1B mutant that harbors an mRFP fused with a P2a ribosome skipping motive to the N terminus of the major tegument protein VP22, termed vVP22-RFP (Fig 6A). Briefly, the mRFP mutagenesis cassette was amplified from pEP-mRFP-in [39] using primers (Table 1) containing a P2a ribosome skipping motive at the 5’-end and homologue sites for the N-terminus of the UL49 gene (VP22) site at both ends. The luciferase cassette was subsequently inserted by mutagenesis using the epPCRfLuc vector. All primers used for mutagenesis and PCR are given in Table 1. All recombinant BAC genomes were confirmed by PCR and Sanger sequencing the entire insertion with its borders (157829–159546 for TK-fLuc, 111058–114394 for VP22-RFP and 157794–158687 for TK-GFP-SHA) (S1 File). The presence and length of the insertions in the recombinant BAC genomes were also verified by restriction length polymorphisms analysis (RFLP), followed by pulsed field electrophoresis (S5 Fig).

**Table 1:** Primers used for the construction of recombinant MDV.

Construct		Sequence (5’ ➔ 3’)
psiCHECK-2_kana	for	GAGCATGTACACATTCGTGACATCTCTAGGGATAACAGGGTAATCGATTT
	rev	GAATGTGTACATGCTCTGGAAGCCCGCCAGTGTTACAACCAATTAACC
vTK-fLuc insertion of fLuc	for	CACTTCGCATATTAAGGTGACACGCGCGGCCTCGAACACAGCTGCAGGCCATGGCCGATGCTAAGAACATTAAGAAGGG
	rev	ACAAGTTAACGTCGACCCGGGTACCTCTAGATCCGCTAGCGCTTTACTTGTTACACGGCGATCTTGCCGCCTTTCTTAG
vVP22-RFP insertion of mRFP	for	GCAGGAATCGAGACGTCATCATATGCAGAGGGATATCCAAGGGAACGACGCCGTTCCGATTTCCGCCTTTCAGAATCCCC*AGGTCCAGGGTTCTCCTCCACGTCTCCAGCCTGCTTCAGCAGGCTGAAGTTAGTAGCTCCGCTTCC*CAAGGCGCCGGTGGAGTGGC
	rev	CATATGTTGGAGACGCCACACGGTACAATAGAAGGTGCACTTGTTCATATCTTACTGTTTAATATTATATCTTAGTTATCATGGCCTCCTCCGAGGACGT
vVP22-RFP insertion of fLuc	for	TTGAAGCGCATGAACTCCTTGATGACGTCCTCGGAGGAGGCCATGGGCCCCGGATTCTCT
	rev	TGTTCATATCTTACTGTTTAATATTATATCTTAGTTATCATGGCCGATGCTAAGAACAT
vTK-GFP-SHA insertion of SHA	for	agttcgtgaccgccgccgggatcactctcggcatggacgagctgtacaagCCGTCAAGGCCGCATTAC
	rev	acaagttaacgtcgacccgggtacctctagatccgctagcgctttacttgTTAcacggcgatcttgccgcctttcttag

Recombinant viruses were reconstituted by transfection of recombinant MDV BAC DNA into primary CESCs or CECs using an Amaxa nucleofector or the calcium-phosphate method respectively [23, 42]. After 4 to 5 days, virus plaques were detectable, and the viruses were passaged 2–3 times to produce high titer virus stocks that were stored in liquid nitrogen. The parental BAC-derived virus rRB-1B (also named herein WT) served as a positive control for the *in vitro* experiments.

### Plaque size assays

The size of plaques was determined as described previously [43, 44]. CECs or CESCs were seeded in 6-well plates and infected with 100 pfu of indicated viruses. Four or six days later, cells were fixed with 4% paraformaldehyde, permeabilized, blocked and stained with either a cocktail of monoclonal antibodies (anti-gB, ICP4, VP22) or a polyclonal MDV-antiserum. At least 50 plaques were imaged and plaques sizes determined using the AxioVision (Zeiss) or Image J software (NIH).

### Multi-step growth kinetics

Plaque size data were confirmed by qPCR-based multi-step growth kinetics as described previously [45]. Briefly, one million CECs or CESCs were infected with 100 pfu of the respective viruses and virus replication assessed by qPCR (see below) over 5 or 6 days of infection.

### Animal experiments

Specific pathogen-free WL chickens (B13/B13 haplotype) (named WL B13) were obtained from the PFIE animal experimental platform, INRAE Centre Val de Loire. Chickens were fed with “pullet starter”, the composition of which is available on request.

#### Pilot experiment

An anesthetized uninfected 8-day-old chicken was imaged with the procedure described below to estimate the luminescence background of the body. One anesthetized 10-day-old chicken was inoculated with vTK-fLuc in two locations: 0.1 mL intramuscularly into the breast muscle and 0.1mL subcutaneously at the abdomen above the cloaca. Next, the *in vivo* imaging procedure was applied (see below), with three images acquired at 7, 10 and 15 min after D-luciferin injection. After each experiment, the chickens were humanely euthanized by pentobarbital injection under anesthesia.

#### Experiment 1

Thirteen one-day-old chickens were inoculated intramuscularly with 2000 pfu (0.1mL) of vTK-fLuc, whereas a control group (CTL) of 3 chickens remained uninfected. Infected birds were housed in an isolator for 14 days. Live imaging was performed using an IVIS Spectrum on six chickens at 7 dpi (#1, 2, 3, 4, 5, 6), on seven chickens at 10 dpi (# 8, 9, 10, 11, 12, 13, 14) and on eight chickens at 14 dpi (#1, 4, 6, 10, 11, 12, 13, 14). The *in vivo* imaging process is described below. To image the internal organs, indicated chickens were humanely euthanized by pentobarbital injection at day 7 pi (# 2, 3, 5), 10 pi (# 8, 9) and 14 pi (#1, 4, 6, 10, 11, 12, 13, 14) under anesthesia. On day 14, food was removed in the early morning, prior to the imaging process. At each time point, one CTL chicken was *in vivo* imaged and euthanized. Upon euthanasia, thymus, bursa, spleen and feathers were collected for *in situ* imaging (bioluminescence), *in vitro* luciferase activity measurement and MDV genome quantification by qPCR. At 14 dpi, skin of feet and toes were also collected to quantify the viral loads by qPCR.

#### Experiment 2

Seven one-day-old chickens were inoculated intramuscularly with 2000 pfu (0.1mL) of vVP22-RFP. Infected birds were housed in an isolator for 14 days. On day 10 pi, blood and feathers were collected to verify infection by qPCR. On day 14 pi, all chickens were humanely euthanized as above. Thymus, bursa, spleen and feathers were collected for MDV genome quantification by qPCR. In addition, both wings and washed feet were harvested and frozen at -80°C for subsequent *ex vivo* imaging by fluorescence.

#### Experiment 3

Six five-day-old chickens were inoculated intramuscularly in the breast muscle with 3000 pfu (0.1mL) of vTK-GFK SHA. Four chickens served as uninfected “contacts” for natural infection via the respiratory route. The ten chickens were housed in the same isolator. A control group of 4 uninfected chickens was housed separately in the SPF animal facility. On day 10 pi, blood and feathers were collected in the inoculated group to verify infection by qPCR. On day 14, four inoculated chickens were humanely killed as described above, while two birds remained as shedders. Wings, feet and the animals’ heads were dissected for immediate in-situ imaging by fluorescence. On day 31 pi, blood and feathers were collected from the remaining birds to verify infection by qPCR. On day 35 pi, the two remaining inoculated birds were humanely killed, necropsied and body parts were collected and imaged as at 14 dpi. On day 44 pi, blood and feathers were collected from the contact birds to verify naturally transmitted infection. On day 49 pi, the four contact birds were humanely killed, necropsied, and body parts were collected and imaged.

### *In vivo* imaging

Bioluminescence imaging was performed using the *In Vivo* Imaging System IVIS Spectrum (PerkinElmer, Waltham, MA). Each chick was imaged individually according to the following steps: (i) The chicken was weighed. (ii) The chicken was inoculated subcutaneously with the appropriate quantity of D-luciferin substrate (150μg/g of weight with a stock solution at 40mg/mL in PBS). (iii) The chicken was anesthetized using an isoflurane mask device in an impervious box (Perkin Elmer, Massachusetts, USA) and subsequently placed on its back, splayed wings on the IVIS Spectrum cabinet platform. Special attention was paid to the removal of feces on the edge of cloaca if present. (iv) Imaging was performed starting 10 min after D-luciferin injection. Of note, the D-Luciferin kinetic curve for luciferase activity was previously verified in our model. Isoflurane anesthesia (1.5 l/min O2, 3% isoflurane) was maintained over the course of imaging through a beak’s cone. (v) Each image was first acquired with the automatic mode (2 min being the maximum of exposure time); When the signal was low, image acquisition was performed with the manual mode for 5 min exposure. (vi) The chicken was placed in a box until it woke up and subsequently either reintroduce into the isolator or directly euthanized still under anesthesia. At each time point, one age-matched control chicken was imaged. Bioluminescence data were uniformly acquired and analyzed with the PerkinElmer Living Image software (version 4.2, Perkin Elmer). Note that all tags on images are in total flux and all scales in radiance, as provided by the software. When the signal was quantified by zones (in average radiance or total flux) and plotted, the threshold of detection of bioluminescence was defined as the mean plus 2 standard deviations of the bioluminescence of the control chickens for the same zones.

### *Ex vivo* imaging of organs and imaging virus-infected cells in cell culture

CESCs infected with the vTK-fLuc in cell culture were assayed for a bioluminescence signal as follows: the supernatant was removed, a D-luciferin solution diluted in PBS (at 2 or 4 mg/ml) was added and the plates were imaged in the IVIS Spectrum. For the proportionality assay, a D-luciferin solution at 2mg/ml was used, with 200μl per well of 24-well plate. For infected CESCs in suspension, prepared as an inoculum: cells were thawed and 100μl of the cell suspension (at 4000 pfu or 2000 pfu) was mixed with 50μl of D-luciferin at 4mg/ml.

In experiment 1, at each time point and for each euthanized chick, four to five thymus lobes, half a bursa, half a spleen and feather material from 4–5 wing growing feathers (live feather epithelium and pulp) were freshly collected and placed in a petri dish, finely chopped and immersed in D-Luc substrate (200μl at 4.5mg/mL in PBS) and immediately imaged with the IVIS Spectrum for bioluminescence. Bioluminescence data were uniformly acquired and analyzed as described above.

In experiment 2, wings and feet were collected from rVP22-RFP-infected chickens at necropsy and frozen at -80°C. Note that the feet were rinsed with water to remove dirt before freezing. The fluorescence imaging was performed on the “freshly” thawed samples (4 days of freezing). Detection of the RFP signal was performed using the spectral unmixing mode acquisition and analysis function of the IVIS Spectrum. Two simultaneous acquisition conditions with automatic mode were applied: (i) excitation at 535 nm (bandwidth of 30 nm), and emission at 580-600-620-640 nm (bandwidths of 20 nm), and (ii) excitation at 570 nm (bandwidth of 30 nm), and emission at 620-640-660-680 nm (bandwidths of 20 nm). Spectral unmixing analysis was performed by Living Image 4.7 software. RFP signals and tissue autofluorescence signals were assessed. The fluorescence quantification of several ROIs was performed on the image "RFP signal” minus “tissue autofluorescence signal". The results were expressed in Average Radiant Efficiency [p / s / cm^2^ / sr] / [μW / cm^2^].

In experiment 3, wings, feet and heads were collected from rTK GFP-SHA-infected chickens and imaged “fresh”. Detection of the GFP signal was performed using the spectral unmixing mode acquisition and analysis function of the IVIS Spectrum. Two simultaneous acquisition conditions with automatic mode were applied: (i) excitation at 465 nm (bandwidth of 30 nm), and emission at 520-540-560-580 nm (bandwidths of 20 nm).

### *In vitro* luciferase activity

At each time point, each piece of fresh organs harvested (feather material, spleen, bursa, thymus) was weighed (50-90mg for feather material, 40-80mg for spleen, 50-100mg for bursa, 90-140mg for thymus), homogenized and lysed mechanically with a micropotter in a 1.5mL tube using 300μL of Cell Culture Lysis Reagent. Luciferase activity was measured using luciferase assay reagent (#E1500, Promega) and a GloMax-Multi Detection System (Promega).

### DNA extraction from tissues and organs

The feather tips material collection and subsequent DNA extraction was performed using the QiaAmp DNA kit as previously described [46]. The skin of the feet (metatarsus and dorsal part of the toes) was harvested, finely chopped and the DNA extracted as for feathers material. For blood, PBMCs were first isolated using density-gradient centrifugation by MSL (Eurobio). DNA was next extracted from PBMCs as previously described using the QiaAmp DNA kit [33]. DNA from 20 mg of dust collected from the isolator was extracted as previously described [23].

### Isolation of keratinocytes from feet skin covered of scales and infectivity assay

Chicken feet were disinfected with iodine and 70% ethanol. Skin fragments were excised and incubated with 0.5 mg/mL thermolysin (#T7902-100MG, Sigma) either 2 h at 37°C for 19-day old chickens or overnight at 4°C for older chickens. After its separation from the dermis, the epidermis was enzymatically digested 10 min at 37°C in a 0.05% trypsin-EDTA solution (#11580626, Gibco). Isolated chicken primary keratinocytes (CPKs) were filtered through a 40 μm mesh strainer (Falcon) and centrifuged 7 min at 1400 rpm. The CPKs pellet was resuspended in 3 mL William’s E medium (#32551–020, Gibco) supplemented with 3% chicken serum, 2% fetal calf serum, 1% L-glutamine and 1% penicillin-streptomycin (#DE17-602E, Lonza).

For the infectivity assay, 1 mL of the CPK suspension was added on two wells of a 6-well plate containing a confluent layer of CESCs. After an overnight co-culture, cells were washed with PBS and then cultivated 3 to 4 days with William’s E medium (#32551–020, Gibco) supplemented with 1.5% chicken serum, 1% fetal calf serum, 1% L-glutamine and 1% penicillin-streptomycin (#DE17-602E, Lonza).

### Immunostaining and fluorescence microscopy on isolated chicken primary keratinocytes

1ml of CPKs were fixed with 1% paraformaldehyde at 4°C for 24 hrs and rinsed with PBS. CPKs were plated by centrifugation at low speed (500 × g; 800rpm) for 5 min on 0.17μm-thick- glass coverslips using a Cytospin 4 devise (ThermoFisher Scientific). For MDV antigens, the immunostainings were performed in a blocking solution (PBS, 0.1% Triton X-100, 1% bovine serum albumin) for 1 h at room temperature, then incubated for 45 min with a GAM secondary antibody coupled to Alexa Fluor 594 and finally nuclei counterstaining with Hoechst 33342 dye (1:2000e) (Invitrogen) and mounting with Mowiol (Merck). Samples were assessed using an Axiovert 200 M inverted epifluorescence microscope equipped with an Apotome imaging system (Zeiss). Images were captured with an AxioCam MRm charge-coupled-device camera (Zeiss) using the Axiovision software (Zeiss).

### Immunostaining of feet skin cryosections by confocal microscopy

Skin samples were snap-frozen in nitrogen-cooled isopentane. A polyclonal anti-GFP primary antibody (1:1000; Abcam, Cambridge, MA, United States) was used for immunostaining. Briefly, 10 μm-thick sections were incubated in a blocking solution (Diagomics, Blagnac, France) for 30 min at room temperature (RT) and then incubated with the primary antibody overnight at 4°C. A specific secondary Alexa Fluor 555-coupled antibody (1:300; Invitrogen, Karlsruhe, Germany) was then used for 1 hour at RT before PBS washing, nuclei counterstaining with DAPI (1:1000; BioStatus Ltd, Shepshed, UK) and mounting using Mowiol medium. Samples were observed with a spectral confocal microscope LSM 780 (Zeiss, Oberkochen, Germany).

### Quantification of MDV genome copies by quantitative (qPCR)

To assess MDV genome copy numbers in the samples described above, we performed qPCR as described previously [33, 47]. Briefly, copy numbers were quantified by detecting the viral gene ICP4. The number of cells in each sample was assessed by quantifying the copy number of the cellular iNOS gene. For each sample, the MDV genome copy number was calculated per million cells.

### Statistical analysis

Statistical analyses were performed using Graph-Pad Prism v7 (GraphPad Software, Inc., USA) and the SPSS software (SPSS Inc., USA). The multi-step growth kinetics were analyzed with the Kruskal-Wallis test. Analysis for plaque size assays were analyzed with the Mann-Whitney test. For the correlation analyses, the Spearman test was used. GraphPad Prism v7 was used for plots and computing. The bioluminescence and fluorescence of organs *ex vivo* was analyzed by a Kruskal-Wallis test with a Dunn correction for multiple comparison.

## Supporting information

S1 Fig Establishment of the parameters for bioluminescence in vivo imaging in chickens. Herein, all measures of bioluminescence indicated on images in red are in average radiances in p/s/cm2/sr. A. Food auto-luminescence. Two grams of « pullet starter » granules. B. Chicken auto-luminescence. An 8-day-old chick was injected with D-Luc subcutaneously and imaged live 10 min later. An average radiance of 1.281x10^3^p/s/cm2/sr was measured from a ROI on the abdomen. C. Evaluation of imaging time after D-Luc inoculation. A 10-day-old chicken was inoculated with vTK-fLuc (about 10^4^ pfu) in two locations (intramuscularly into the breast and subcutaneously at the abdomen level above the cloaca), injected with D-Luc subcutaneously and imaged live 7, 10 and 15 min later.
(TIF)

S2 Fig Bioluminescence *in vivo* imaging of MDV vTK-fLuc-infected chickens using IVIS spectrum. Nine ROIs were defined in order to quantify the bioluminescent signals in beak, wings (flight feathers), feet (covered of scales), upper chest (anatomical site of the thymus), lower abdomen (anatomical site of the bursa) and thigh (as a second feathered zone, with body feathers). Images are shown at 7 dpi (A), 10 dpi (B) and 14 dpi (C) with an age-matched control chicken. Some wings ROI are not shown at 14 dpi, because overlapping with thighs or upper chest ones.
(TIF)

S3 Fig Relationship between the fLuc reporter activity and the viral loads *in vivo*. The bioluminescence (in total flux) of both wings at 7, 10 and 14 dpi were added and compared to the viral loads determined in the feathers of these wings (n = 18). A correlation analysis was performed through Spearman test. The result supports a significant and positive correlation between the two measures (rho = 0.9443, p-value<0.0001).
(TIF)

S4 Fig Fluorescence ex vivo imaging of MDV vTK-GFP-SHA-infected chickens. A. Images of the feet of four infected chicken at 14 dpi and two age-matched control chickens using IVIS spectrum. B. Images of the feet of two infected chickens at 35 dpi and one age-matched control chicken using IVIS spectrum. C. Images of the beak and claws of one infected chicken at 35 dpi and one age-matched control chicken using a Leica fluorescent stereomicroscope MZ10F. Images were captured with DFC3000 monochrome camera (Leica) by using the LAS X software (Leica). Chicken #83 is shown as an example of the green fluorescent signal observed in both animals.
(TIF)

S5 Fig Analyses of the recombinant virus genomes by RFLP. RFLP analyses of the indicated recombinant BAC clones were performed using *Eco*RI. The predicted "theoretical" (left panel) and "experimental" (right panel) profiles are shown. The predicted "theoretical" digestion profiles were obtained with pDRAW32 software (v1.1.147). (A) The wild type RB-1B BAC, two TK-GFP-SHA clones (10.3 was used in this study) and the TK-fLuc clone were digested with *Eco*RI and resolved on an agarose gel for 4 h. (B) The parental RB-1B clone, the intermediate and final clone of the VP22-RFP BAC were digested with *Eco*RI and resolved on an agarose gel for 16 h. Red arrows indicated expected changes in the RFLP profile.
(TIF)

S1 File Results of Sanger sequencing on each mutant virus insert. (DOCX)
