# Supplementary figures and images for: In vivo imaging reveals novel replication sites of a highly oncogenic avian herpesvirus in chickens
Source: PLoS Pathog. 2022 Aug 29;18(8):e1010745. doi: 10.1371/journal.ppat.1010745 (PMC9462805; doi:10.1371/journal.ppat.1010745)

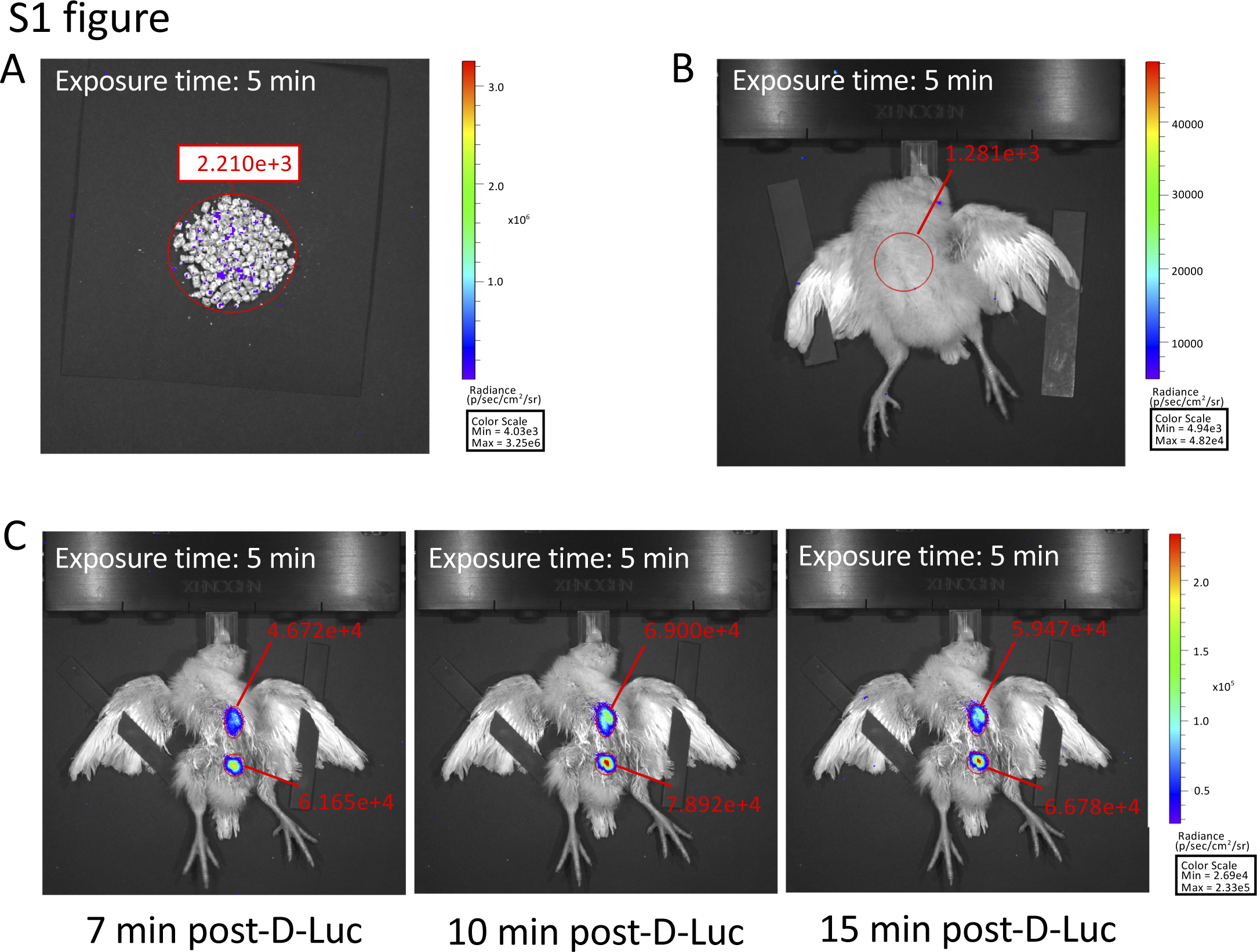

Supplement: S1 Fig — Herein, all measures of bioluminescence indicated on images in red are in average radiances in p/s/cm2/sr. A. Food auto-luminescence. Two grams of « pullet starter » granules. B. Chicken auto-luminescence. An 8-day-old chick was injected with D-Luc subcutaneously and imaged live 10 min later. An average radiance of 1.281x10^3^p/s/cm2/sr was measured from a ROI on the abdomen. C. Evaluation of imaging time after D-Luc inoculation. A 10-day-old chicken was inoculated with vTK-fLuc (about 104 pfu) in two locations (intramuscularly into the breast and subcutaneously at the abdomen level above the cloaca), injected with D-Luc subcutaneously and imaged live 7, 10 and 15 min later. (TIF) [file ppat.1010745.s001.tif]

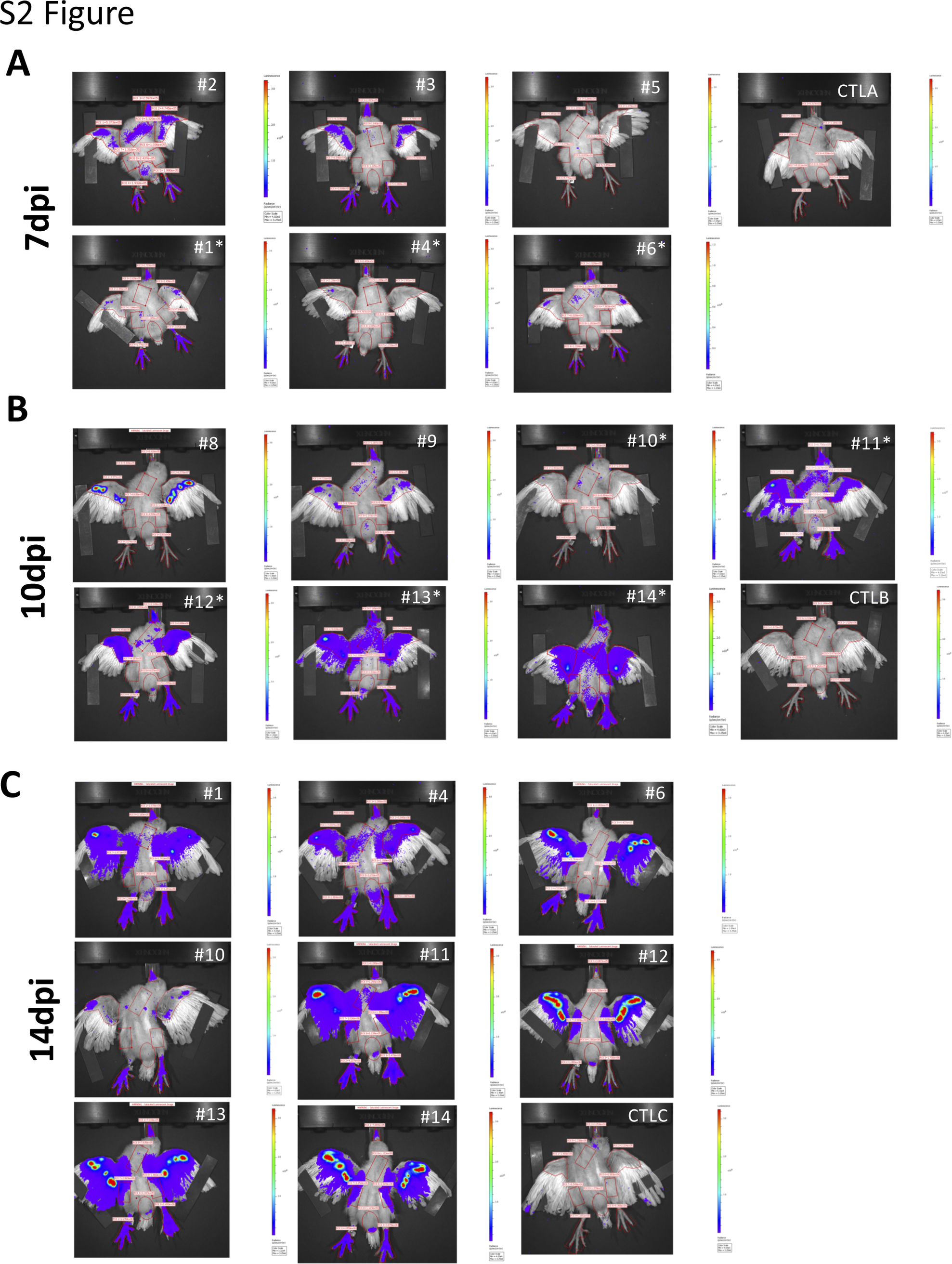

Supplement: S2 Fig — Nine ROIs were defined in order to quantify the bioluminescent signals in beak, wings (flight feathers), feet (covered of scales), upper chest (anatomical site of the thymus), lower abdomen (anatomical site of the bursa) and thigh (as a second feathered zone, with body feathers). Images are shown at 7 dpi (A), 10 dpi (B) and 14 dpi (C) with an age-matched control chicken. Some wings ROI are not shown at 14 dpi, because overlapping with thighs or upper chest ones. (TIF) [file ppat.1010745.s002.tif]

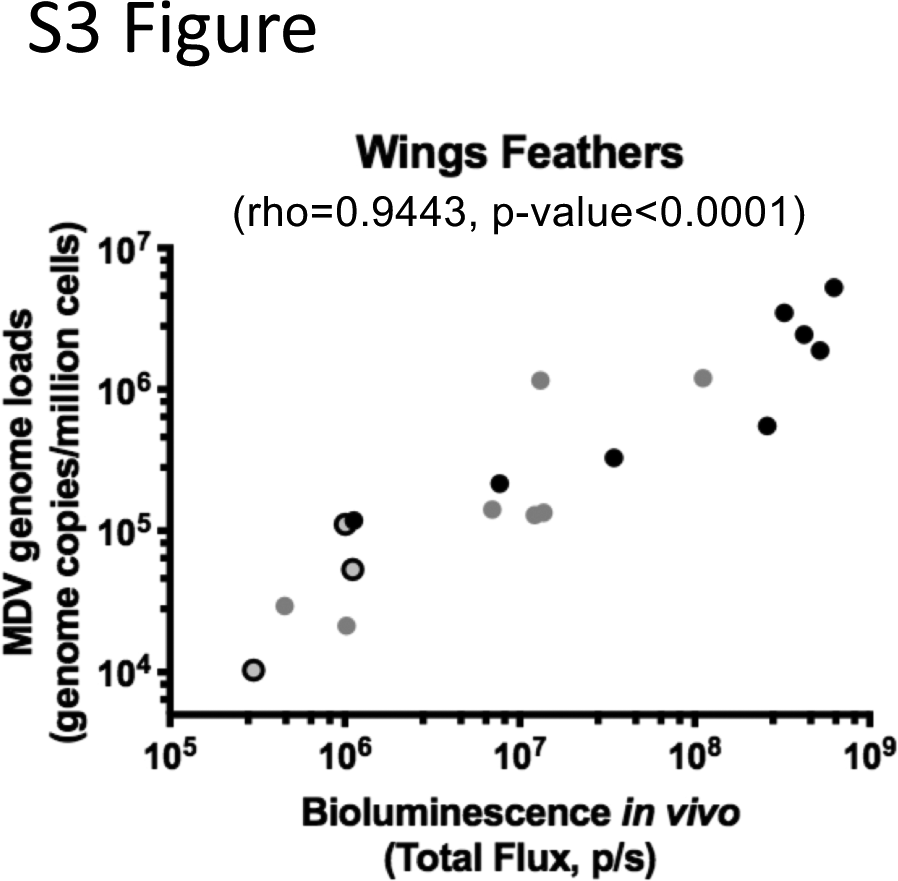

Supplement: S3 Fig — The bioluminescence (in total flux) of both wings at 7, 10 and 14 dpi were added and compared to the viral loads determined in the feathers of these wings (n = 18). A correlation analysis was performed through Spearman test. The result supports a significant and positive correlation between the two measures (rho = 0.9443, p-value<0.0001). (TIF) [file ppat.1010745.s003.tif]

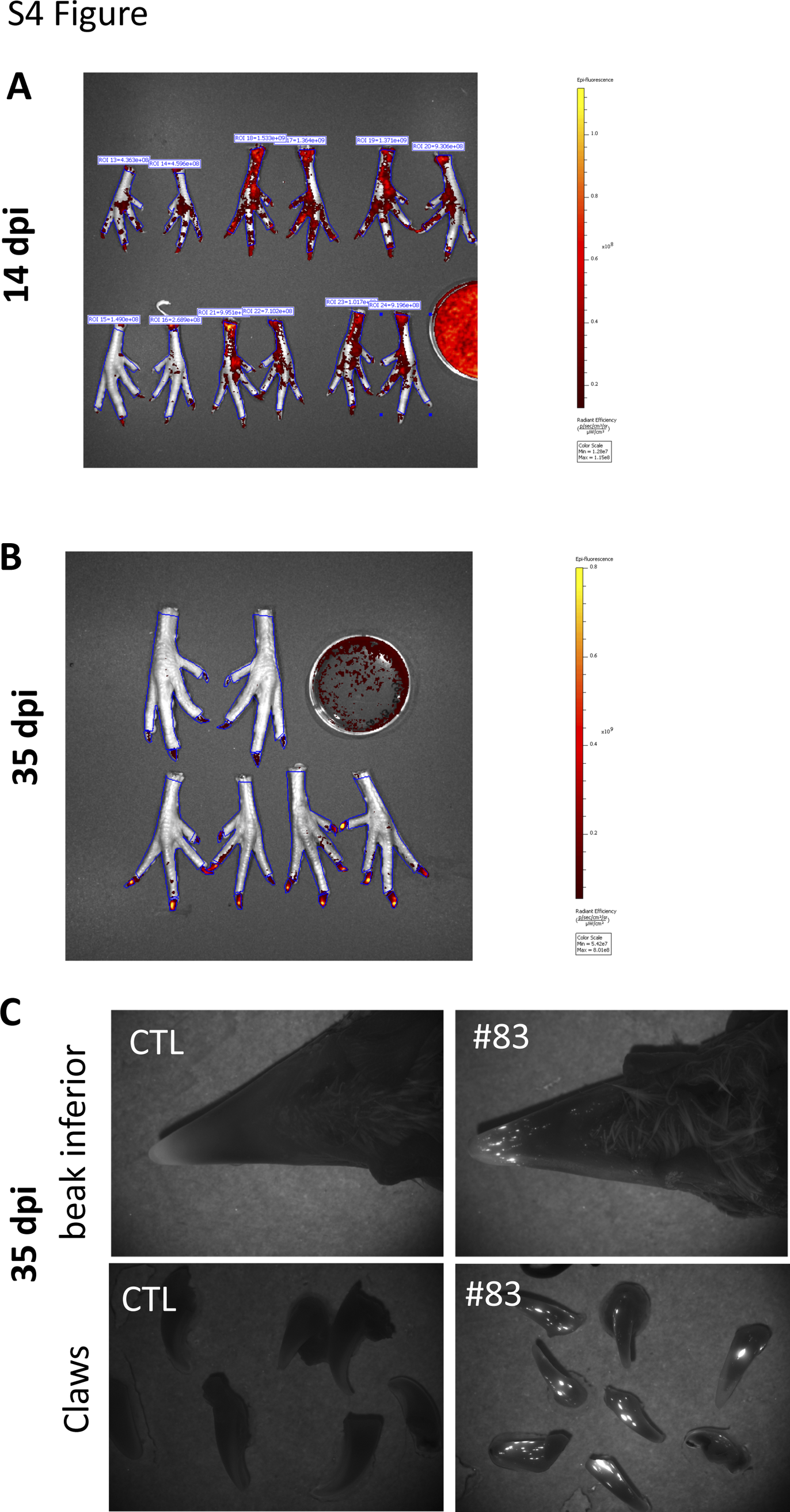

Supplement: S4 Fig — A. Images of the feet of four infected chicken at 14 dpi and two age-matched control chickens using IVIS spectrum. B. Images of the feet of two infected chickens at 35 dpi and one age-matched control chicken using IVIS spectrum. C. Images of the beak and claws of one infected chicken at 35 dpi and one age-matched control chicken using a Leica fluorescent stereomicroscope MZ10F. Images were captured with DFC3000 monochrome camera (Leica) by using the LAS X software (Leica). Chicken #83 is shown as an example of the green fluorescent signal observed in both animals. (TIF) [file ppat.1010745.s004.tif]

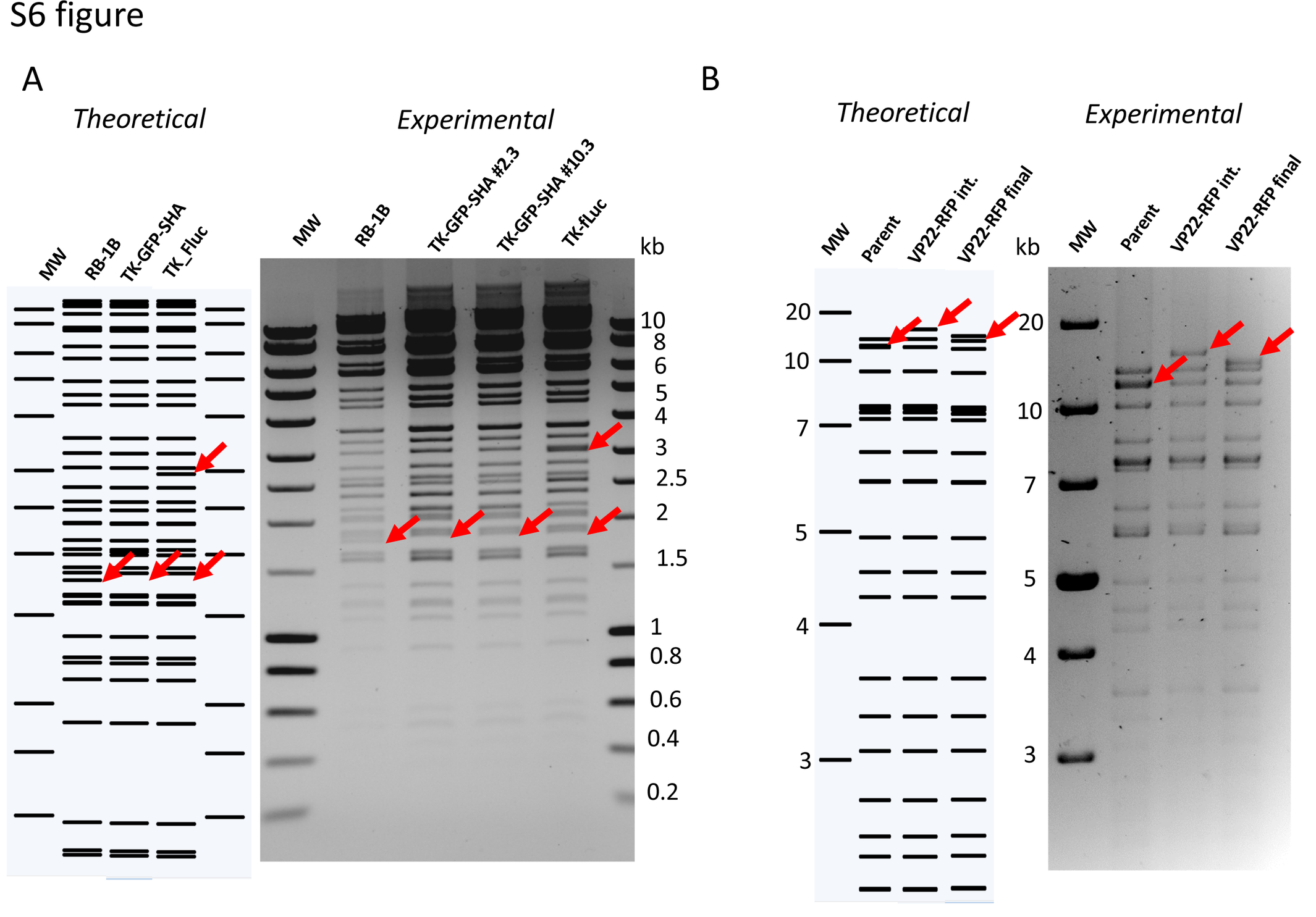

Supplement: S5 Fig — RFLP analyses of the indicated recombinant BAC clones were performed using EcoRI. The predicted "theoretical" (left panel) and "experimental" (right panel) profiles are shown. The predicted "theoretical" digestion profiles were obtained with pDRAW32 software (v1.1.147). (A) The wild type RB-1B BAC, two TK-GFP-SHA clones (10.3 was used in this study) and the TK-fLuc clone were digested with EcoRI and resolved on an agarose gel for 4 h. (B) The parental RB-1B clone, the intermediate and final clone of the VP22-RFP BAC were digested with EcoRI and resolved on an agarose gel for 16 h. Red arrows indicated expected changes in the RFLP profile. (TIF) [file ppat.1010745.s005.tif]
