# Supplementary material for: In vivo imaging reveals novel replication sites of a highly oncogenic avian herpesvirus in chickens
Source: PLoS Pathog. 2022 Aug 29;18(8):e1010745. doi: 10.1371/journal.ppat.1010745 (PMC9462805; doi:10.1371/journal.ppat.1010745)
Supplement: S1 File — (DOCX) [file ppat.1010745.s006.docx]

**S1 File: Results of Sanger sequencing on each mutant virus insert**

**TK-fLuc mutant**

- Border sequences of each reporter gene are underlined
- The start and stop of reporter gene indicated in bold and capital letters
- Silent mutations are highlighted in blue; non-silent mutations are in red.

(157829)acacagctgcaggcc**ATG**gccgatgctaagaacattaagaagggccctgctcccttctaccctctggaggatggcaccgctggcgagcagctgcacaaggccatgaagaggtatgccctggtgcctggcaccattgccttcaccgatgcccacattgaggtggacatcacctatgccgagtacttcgagatgtctgtgcgcctggccgaggccatgaagaggtacggcctgaacaccaaccaccgcatcgtggtgtgctctgagaactctctgcagttcttcatgccagtgctgggcgccctgttcatcggagtggccgtggcccctgctaacgacatttacaacgagcgcgagctgctgaacagcatgggcatttctcagcctaccgtggtgttcgtgtctaagaagggcctgcagaagatcctgaacgtgcagaagaagctgcctatcatccagaagatcatcatcatggactctaagaccgactaccagggcttccagagcatgtacacattcgtgacatctcatctgcctcctggcttcaacgagtacgacttcgtgccagagtctttcgacagggacaaaaccattgccctgatcatgaacagctctgggtctaccggcctgcctaagggcgtggccctgcctcatcgcaccgcctgtgtgcgcttctctcacgcccgcgaccctattttcggcaaccagatcatccccgacaccgctattctgagcgtggtgccattccaccacggcttcggcatgttcaccaccctgggctacctgatttgcggctttcgggtggtgctgatgtaccgcttcgaggaggagctgttcctgcgcagcctgcaagactacaaaattcagtctgccctgctggtgccaaccctgttcagcttcttcgctaagagcaccctgatcgacaagtacgacctgtctaacctgcacgagattgcctctggcggcgccccactgtctaaggaggtgggcgaagccgtggccaagcgctttcatctgccaggcatccgccagggctacggcctgaccgagacaaccagcgccattctgattaccccagagggcgacgacaagcctggcgccgtgggcaaggtggtgccattcttcgaggccaaggtggtggacctggacaccggcaagaccctgggagtgaaccagcgcggcgagctgtgtgtgcgcggccctatgattatgtccggctacgtgaataaccctgaggccacaaacgccctgatcgacaaggacggctggctgcactctggcgacattgcctactgggacgaggacgagcacttcttcatcgtggaccgcctgaagtctctgatcaagtacaagggctaccaggtggccccagccgagctggagtctatcctgctgcagcaccctaacattttcgacgccggagtggccggcctgcccgacgacgatgctggcgagctgcctgccgccgtcgtcgtgctggaacacggcaagaccatgaccgagaaggagatcgtggactatgtggccagccaggtgacaaccgccaagaagctgcgcggcggagtggtgttcgtggacgaggtgcccaagggcctgaccggcaagctggacgcccgcaagaaccgcgagatcctgatcaaggctaagaaaggcggcaagatcgccatg**TAA**caagtaaagcgctagcggatctagaggtacccgggtcgacgttaacttgt(159546)

The sequencing revealed that the fLuc insertion was in the correct position. Two non-coding mutations were detected in the fLuc sequence and one non-silent mutation, changing the AA535, from Asn to Ile. The three mutations were detected directly on the BAC and from a PCR amplicon cloned in pJET. Importantly, despite the non-silent mutation, the fLuc was functional in infected cells and birds.

**TK-EGFP-SHA mutant (clone 10.3)**

- Border sequences of each reporter gene are underlined
- GFP sequence indicated in green
- SHA sequence indicated in violet
- Linkers in capital letters
- The start and stop of reporter gene indicated in bold and capital letters

(157794)cacttcgcatattaaggtgacacgcgcggcctcgaacacagctgcaggcc**ATG**gtgagcaagggcgaggagctgttcaccggggtggtgcccatcctggtcgagctggacggcgacgtaaacggccacaagttcagcgtgtccggcgagggcgagggcgatgccacctacggcaagctgaccctgaagttcatctgcaccaccggcaagctgcccgtgccctggcccaccctcgtgaccaccctgacctacggcgtgcagtgcttcagccgctaccccgaccacatgaagcagcacgacttcttcaagtccgccatgcccgaaggctacgtccaggagcgcaccatcttcttcaaggacgacggcaactacaagacccgcgccgaggtgaagttcgagggcgacaccctggtgaaccgcatcgagctgaagggcatcgacttcaaggaggacggcaacatcctggggcacaagctggagtacaactacaacagccacaacgtctatatcatggccgacaagcagaagaacggcatcaaggtgaacttcaagatccgccacaacatcgaggacggcagcgtgcagctcgccgaccactaccagcagaacacccccatcggcgacggccccgtgctgctgcccgacaaccactacctgagcacccagtccgccctgagcaaagaccccaacgagaagcgcgatcacatggtcctgctggagttcgtgaccgccgccgggatcactctcggcatggacgagctgtacaagCCGTCAAGGCCGCATTACCCATACGATGTTCCAGATTACGCTTGGAGCCACCCGCAGTTCGAGAAACTGGGCCTCATGGGC**TAA**agcgctagcggatctagaggtacccgggtcgacgttaacttgt(158687)

**VP22-RFP mutant**

- P2A sequence indicated in yellow
- The start and stop of gene indicated in capital and bold
- Border sequences of the construct are underlined

(111058)tgtactagtttttaattcggatgtctataaaagacgacttacttgcagtagtagggctgttcctatgttaca

**TTA**ttcgctatcactgctacgatatccgcgggcggatgaatgatggcgacgcgaagttgcgggagtgcgagggttatgtg

tacgatgagatcccggatctggcgatttgatttcaatttcaccgccgcgtcttgtacgttcagatttggttgtacgttca

gatttggactttacgttctcgttcccctgggataatccagactcttccaatagtttgcgggcacaggtttcggcttcccc

UL49

catcaaatttggaccctcttgaatggtaattttaatgacagctctggaaagaaatgcatctaattcttcatttgttcgcg

gaggatcttgacgccaaagggcgagcgcgccttggtatgcgtgatattgagccacagttgcaaccgctccgcaaaacata

cgctgattaaatgccactgtatttgatctccaagtgctagatgctgatgaaggagccgtactgaaagcgaatttattact

ttgtacagctctgaccccgggcttggcacggggtttgtttgtaaagccaccccgggtggtagacgaaacgtcttccgata

cggctttcgccgatttatgtttttgtgttggatggtcggtaaatggtccatgttttgacaaatcatcctggtttaaattt

cgctgagtacgtgttgatggtctgcgagcaggaatcgagacgtcatcatatgcagagggatatccaagggaacgacgccg

ttccgatttccgcctttcagaatcCCCaggtccagggttctcctccacgtctccagcctgcttcagcaggctgaagttag

tagctccgcttccCAAggcgccggtggagtggcggccctcggcgcgctcgtactgttccacgatggtgtagtcctcgttg

tgggaggtgatgtccagcttgatgtcggtcttgtaggcgccgggcagctgcacgggcttcttggccatgtaggtggtctt

gacctcggcgtcgtagtggccgccgtccttcagcttcagcctcatcttgatctcgcccttcagggcgccgtcctcggggt

acatccgctcggtggaggcctcccagcccatggtcttcttctgcattacggggccgtcggaggggaagttggtgccgcgc

RFP

agcttcaccttgtagatgaactcgccgtcctgcagggaggagtcctgggtcacggtcaccacgccgccgtcctcgaagtt

catcacgcgctcccacttgaagccctcggggaaggacagcttcaagtagtcggggatgtcggcggggtgcttcacgtagg

ccttggagccgtactggaactgaggggacaggatgtcccaggcgaagggcagggggccgcccttggtcaccttcagcttg

gcggtctgggtgccctcgtaggggcggccctcgccctcgccctcgatctcgaactcgtggccgttcacggagccctccat

gcgcaccttgaagcgcatgaactccttgatgacgtcctcggaggaggcCATgggccccggattctcttccacgtcgccag

cctgcttgagcagggaaaaattagttgcgccagaaccCACggcgatcttgccgcctttcttagccttgatcaggatctcg

cggttcttgcgggcgtccagcttgccggtcaggcccttgggcacctcgtccacgaacaccactccgccgcgcagcttctt

ggcggttgtcacctggctggccacatagtccacgatctccttctcggtcatggtcttgccgtgttccagcacgacgacgg

cggcaggcagctcgccagcatcgtcgtcgggcaggccggccactccggcgtcgaaaatgttagggtgctgcagcaggata

gactccagctcggctggggccacctggtagcccttgtacttgatcagagacttcaggcggtccacgatgaagaagtgctc

gtcctcgtcccagtaggcaatgtcgccagagtgcagccagccgtccttgtcgatcagggcgtttgtggcctcagggttat

fLuc

tcacgtagccggacataatcatagggccgcgcacacacagctcgccgcgctggttcactcccagggtcttgccggtgtcc

aggtccaccaccttggcctcgaagaatggcaccaccttgcccacggcgccaggcttgtcgtcgccctctggggtaatcag

aatggcgctggttgtctcggtcaggccgtagccctggcggatgcctggcagatgaaagcgcttggccacggcttcgccca

cctccttagacagtggggcgccgccagaggcaatctcgtgcaggttagacaggtcgtacttgtcgatcagggtgctctta

gcgaagaagctgaacagggttggcaccagcagggcagactgaattttgtagtcttgcaggctgcgcaggaacagctcctc

ctcgaagcggtacatcagcaccacccgaaagccgcaaatcaggtagcccagggtggtgaacatgccgaagccgtggtgga

atggcaccacgctcagaatagcggtgtcggggatgatctggttgccgaaaatagggtcgcgggcgtgagagaagcgcaca

caggcggtgcgatgaggcagggccacgcccttaggcaggccggtagacccagagctgttcatgatcagggcaatggtttt

gtccctgtcgaaagactctggcacgaagtcgtactcgttgaagccaggaggcagatgagatgtcacgaatgtgtacatgc

tctggaagccctggtagtcggtcttagagtccatgatgatgatcttctggatgataggcagcttcttctgcacgttcagg

atcttctgcaggcccttcttagacacgaacaccacggtaggctgagaaatgcccatgctgttcagcagctcgcgctcgtt

fLuc

gtaaatgtcgttagcaggggccacggccactccgatgaacagggcgcccagcactggcatgaagaactgcagagagttct

cagagcacaccacgatgcggtggttggtgttcaggccgtacctcttcatggcctcggccaggcgcacagacatctcgaag

tactcggcataggtgatgtccacctcaatgtgggcatcggtgaaggcaatggtgccaggcaccagggcatacctcttcat

ggccttgtgcagctgctcgccagcggtgccatcctccagagggtagaagggagcagggcccttcttaatgttcttagcat

cggc**CAT**gataactaagatataatattaaacagtaagatatgaacaagtgcaccttctattgtaccgtgtgg(114394)
